# Supplementary material for: Supercritical CO2-assisted rapid synthesis of covalent organic framework-based electrocatalyst for efficient two-electron oxygen reduction reaction
Source: Nat Commun. 2025 Oct 8;16:8963. doi: 10.1038/s41467-025-64901-1 (PMC12508161; doi:10.1038/s41467-025-64901-1)
Supplement: Supplementary file 1 — Supplementary Information [file 41467_2025_64901_MOESM1_ESM.pdf]

Supplementary Information

Supercritical CO_2-Assisted Rapid Synthesis of Covalent Organic Framework-Based Electrocatalyst for Efficient Two-Electron Oxygen Reduction Reaction

Junqi Song[1,#], Zhiqiang Zhang[2,#], Weiping Li[3,#], Chunli Liu[3], Guodong Feng[3], Yaqiong Su[3], Kai Xi[3,*], Hong Yi[2,*], Changhai Yi[1], Lan Peng[1,*]

[1]Technology Institute, National Engineering Laboratory for Advanced Yarn and Fabric Formation and Clean Production, Wuhan Textile University, Wuhan, 430200, Hubei, P. R. China.

[2]The Institute for Advanced Studies (IAS), Wuhan University, Wuhan 430072, Hubei, P. R. China.

[3]School of Chemistry, Engineering Research Center of Energy Storage Materials and Devices, Ministry of Education, National Innovation Platform (Center) for Industry-Education Integration of Energy Storage Technology, State Key Laboratory of Electrical Insulation and Power Equipment, Engineering Research Center of Energy Storage Material and Chemistry, Xi'an Jiaotong University, Xi'an 710049, Shaanxi, P.R. China.

Correspondence email: lpeng@wtu.edu.cn, hong.yi@whu.edu.cn, kx210.cam@xjtu.edu.cn

Content

1. Experimental section 1

2. Supplementary figures and tables 11

3. Supplementary References 58

1. Experimental section

Materials and reagents

All chemicals were purchased from commercial sources and used without further purification. Potassium hydroxide (KOH, 85%), Potassium ferricyanide ($K_3[Fe(CN)_6]$, 99.9%), Acetic acid (99.8%), Tetrahydrofuran (THF, 99%), Methanol (99.5%) and Acetone (99.5%) were purchased from Sinopharm Chemical Reagent Co., Ltd. (Shanghai, China). Cerium sulfate standard solution ($Ce(SO_4)_2$, 10 mM), Nafion 117 and FAA-3-50 membranes were purchased from Titan Technology Co., Ltd. (Shanghai, China). *n*-Butanol (99.5%) and *o*-Dichlorobenzene (98%) were purchased from Macklin Biochemical Technology Co., Ltd. (Shanghai, China). 2,4,6-Tris(4-formylphenyl)-1,3,5-triazine (TFPT, 97%), Benzenamine, 4,4',4''-(1,3,5-benzenetriyltri-2,1-ethynediyl)tris (TSA, 98%), 4,4',4''-(1,3,5-Triazine-2,4,6-triyl)trianiline (TAPT, 98%), 1,3,5-Tris(4-aminophenyl)benzene (TAPB, 98%) Nafion solution (5 wt%) and Iridium (IV) oxide (IrO_2, 99.9%) were purchased from Aladdin Biochemical Technology Co., Ltd. (Shanghai, China). Multi-walled carbon nanotubes (CNT) were purchased from Xianfeng Nanomaterials Technology Co., Ltd. (Jiangsu, China).

Preparation of SC-COF$_{TSA}$

TFPT (19.6 mg, 0.05 mmol) and TSA (21.2 mg, 0.05 mmol) were mixed and ground. Then, 1.0 mL of *n*-butanol and 0.2 mL of 12 M acetic acid aqueous solution were added. After 5 minutes of sonication, the mixture was put into the reactor. CO_2 was introduced into the reaction setup, and the conditions were maintained at 8 MPa and 80 °C for 1 hour. After the reaction was complete, the pressure in the apparatus was slowly released, and the product was retrieved. The product was collected by filtration and washed several times with methanol and acetone. Soxhlet extraction was performed in a tetrahydrofuran (THF) solution at 90 °C for 6 hours. Finally, the product was dried under vacuum at 80 °C for 12 hours to yield the pure product.

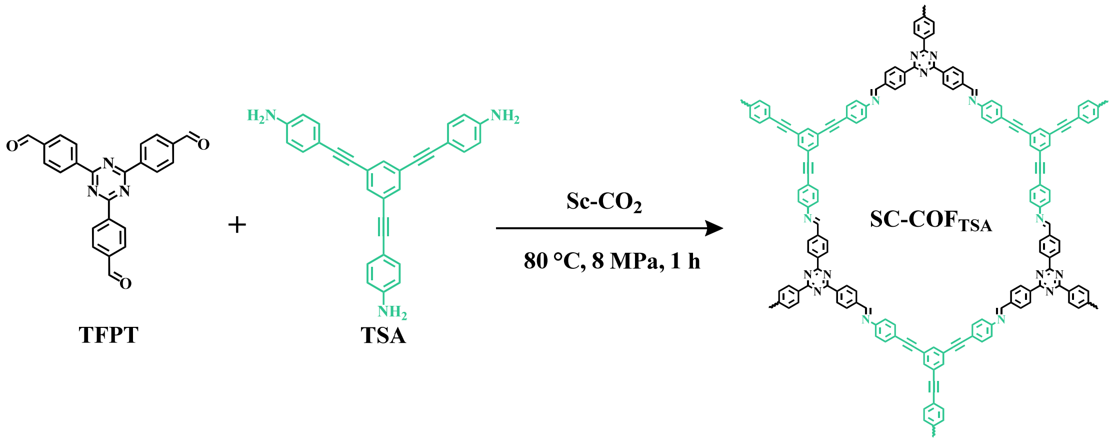

Preparation of SC-COF$_{TAZ}$

TFPT (19.6 mg, 0.05 mmol) and TAPT (17.7 mg, 0.05 mmol) were mixed and ground. Then, 0.4 mL of *n*-butanol and 0.2 mL of 12 M acetic acid aqueous solution were added. After 5 minutes of sonication, the mixture was put into the reactor. CO_2 was introduced into the reaction setup, and the conditions were maintained at 8 MPa and 80 °C for 1 hour. After the reaction was complete, the pressure in the apparatus was slowly released, and the product was retrieved. The product was collected by filtration and washed several times with methanol and acetone. Soxhlet extraction was performed in a tetrahydrofuran (THF) solution at 90 °C for 6 hours. Finally, the product was dried under vacuum at 80 °C for 12 hours to yield the pure product.

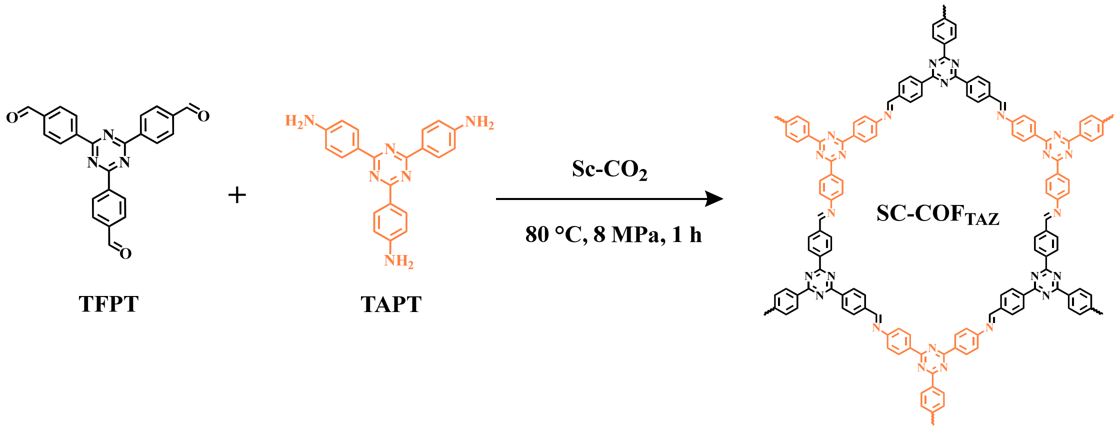

Preparation of SC-COF$_{Ph}$

TFPT (19.6 mg, 0.05 mmol) and TAPB (17.5 mg, 0.05 mmol) were mixed and ground. Then, 0.6 mL of *n*-butanol and 0.2 mL of 12 M acetic acid aqueous solution were added. After 5 minutes of sonication, the mixture was put into the reactor. CO_2 was introduced into the reaction setup, and the conditions were maintained at 8 MPa and 80 °C for 1

hour. After the reaction was complete, the pressure in the apparatus was slowly released, and the product was retrieved. The product was collected by filtration and washed several times with methanol and acetone. Soxhlet extraction was performed in a tetrahydrofuran (THF) solution at 90 °C for 6 hours. Finally, the product was dried under vacuum at 80 °C for 12 hours to yield the pure product.

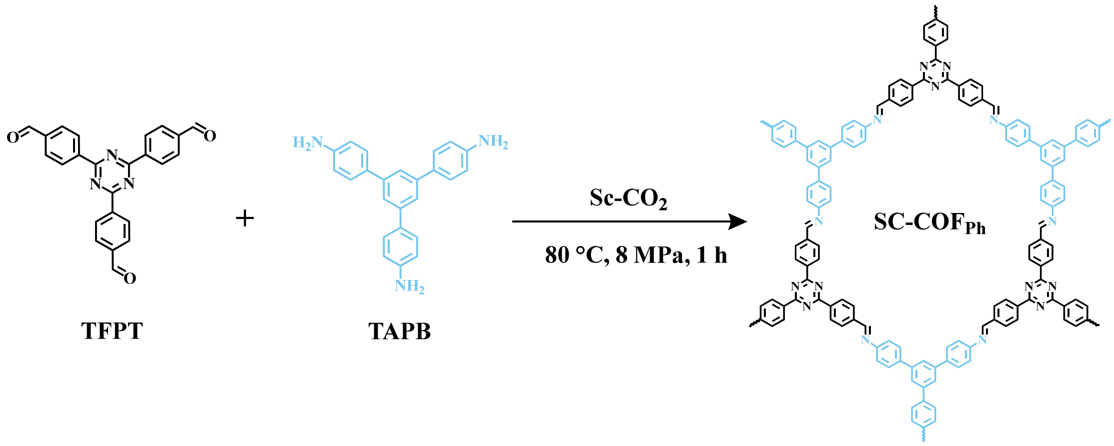

Preparation of SC-COF$_{TSA}$@CNT-X (X=10, 20, 30 and 50)

Following a synthesis method similar to that of SC-COF$_{TSA}$, where X represents the mass ratio of CNT loading to COF monomer (X = $\frac{m_{CNT}}{m_{TFPT}+m_{TSA}}$), we take SC-COF$_{TSA}$ @CNT-30 as an example. TFPT (19.6 mg, 0.05 mmol) and TSA (21.2 mg, 0.05 mmol) were mixed and ground together. Then, CNT (12.24 mg) was added and the grinding continued. After that, 1.0 mL of *n*-butanol and 0.2 mL of 12 M acetic acid aqueous solution were added. The mixture was sonicated for 5 minutes before being placed into the reactor. CO_2 was introduced into the reaction device and the conditions were maintained at 8 MPa and 80 °C for 1 hour. Afterward, the pressure in the device was slowly released, and the product was removed. The product was collected by filtration and extracted with THF at 90 °C for 12 hours using a Soxhlet extractor. Finally, the pure product was obtained by vacuum drying at 80 °C.

Preparation of SC-COF$_{TAZ}$@CNT-30

TFPT (19.6 mg, 0.05 mmol) and TAPT (17.7 mg, 0.05 mmol) were mixed and ground together. Then, CNT (11.19 mg) was added, and the grinding continued. Subsequently, 0.4 mL of *n*-butanol and 0.2 mL of 12 M acetic acid aqueous solution were added. The mixture was sonicated for 5 minutes before being placed into the reactor. CO_2 was introduced into the reaction device, and the conditions were maintained at 8 MPa and

80 °C for 1 hour. Afterward, the pressure in the device was slowly released, and the product was removed. The product was collected by filtration and extracted with THF at 90 °C for 12 hours using a Soxhlet extractor. Finally, the pure product was obtained by vacuum drying at 80 °C.

Preparation of SC-COF$_{Ph}$@CNT-30

TFPT (19.6 mg, 0.05 mmol) and TAPB (17.5 mg, 0.05 mmol) were mixed and ground together. Then, CNT (11.13 mg) was added, and the grinding continued. Next, 0.6 mL of *n*-butanol and 0.2 mL of 12 M acetic acid aqueous solution were added. The mixture was sonicated for 5 minutes before being placed into the reactor. CO_2 was introduced into the reaction device, and the conditions were maintained at 8 MPa and 80 °C for 1 hour. Afterward, the pressure in the device was slowly released, and the product was removed. The product was collected by filtration and extracted with THF at 90 °C for 12 hours using a Soxhlet extractor. Finally, the pure product was obtained by vacuum drying at 80 °C.

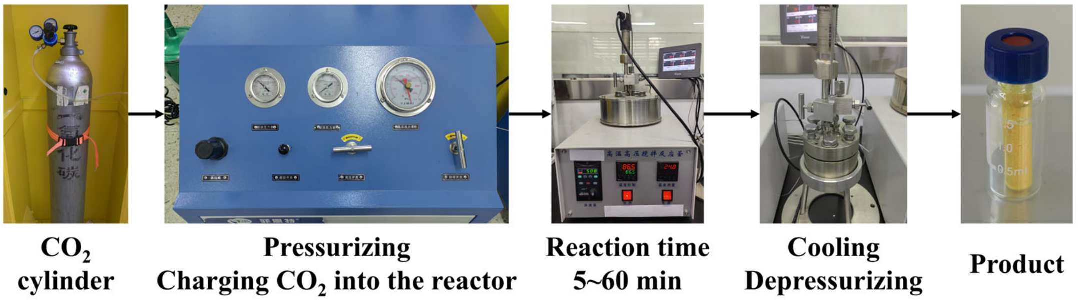

Supplementary Figure 1. Operational flow diagram of supercritical reactor.

Materials characterization

X-ray diffraction (XRD) data were recorded on an Empyrean diffractometer with Cu-Kα radiation by depositing powder on a glass substrate, from 2 Theta = 3° to 35° with a velocity of 10° min^{-1}. Scanning electron micrograph (SEM) images and energy dispersive spectroscopy (EDS) mapping images were recorded with a HITACHI Regulus-8100 microscope (Tokyo, Japan) at an accelerating voltage of 5 kV. Transmission electron microscopy (TEM) images were observed on a JEM-2100 (HT) electron microscope (JEOL, Japan) with an accelerating voltage of 200 kV. Attenuated-total-reflection High-resolution transmission electron microscope (HR-TEM) was

collected on a JEM-2200FS transmission electron microscope (JEOL, Japan) at an operation voltage of 200 kV. Fourier transform infrared spectroscopy (ATR-FTIR) analysis was performed using a Nicolet iS50 FTIR spectrometer (Thermo). N_2 sorption isotherms were measured at 77 K with a TriStar II instrument (Micromeritics). The specific surface area was calculated from the nitrogen adsorption isotherm using the Brunauer-Emmett-Teller (BET) equation and the pore size distribution using the Quenched Solid Density Functional Theory model (QSDFT equilibrium model). Infrared spectroscopy was carried out using a Fourier transform infrared (FT-IR) spectrometer (SHIMADZU IRTracer-100). Solid-state ^{13}C NMR data of catalysts were recorded with Bruker AVANCE NEO 400 MHz spectrometers. Thermogravimetric analysis (TGA) was conducted on a TG 209 F1 Instruments by heating COF from 20 to 800 ℃ with 10 ℃ min^{-1} in N_2 atmosphere. Solid state ultraviolet-visible diffuse reflectance absorption spectra (UV-Vis-DRS) were collected using a UV3600 spectrophotometer (SHIMADZU, Japan).

Structural modeling and powder X-ray diffraction (PXRD) refinement of SC-COF

Structural modeling of SC-COF was conducted using the *Materials Studio* software package. The model was constructed in an initial lattice with the space group *P*1, and geometric optimization of the proposed model was performed using the MS Forcite molecular dynamics module to obtain optimized lattice parameters. The *P*6 space group was applied to the AA stacking model, and the AB stacking structure of SC-COF was investigated by offsetting the stacking units from the AA model. Results showed that the simulated PXRD pattern of the AA stacking model exhibited the best agreement with the experimental spectrum. The XRD diffraction data and COF model were imported, and Pawley refinement was performed using the *Refinement* function in the Reflex module.

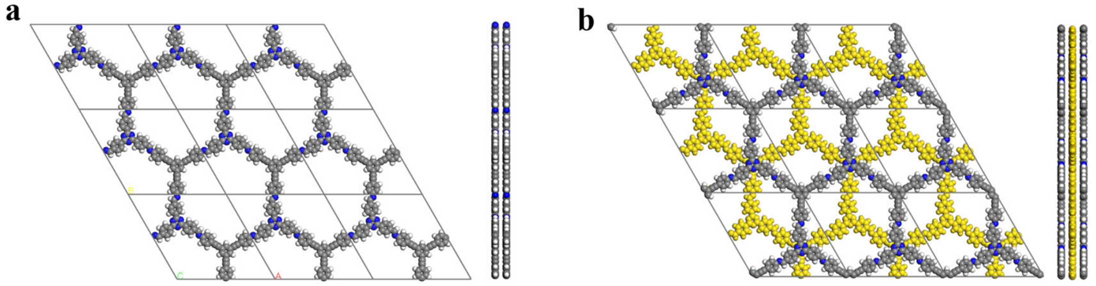

Supplementary Figure S2. The view of the simulated AA-stacking (a) and staggered AB-stacking (b) model of SC-COF$_{TSA}$.

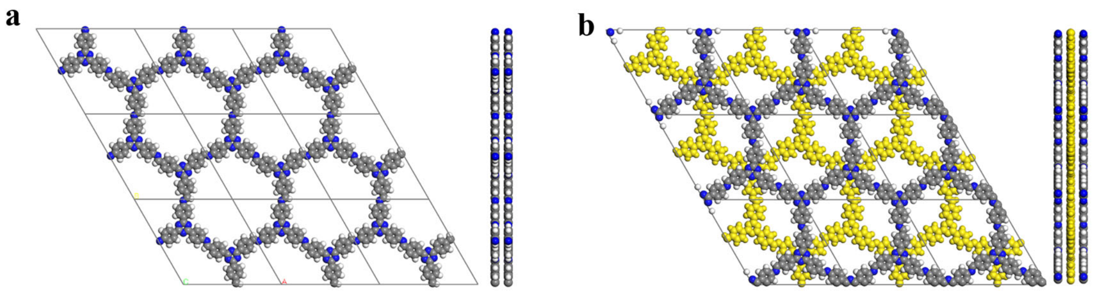

Supplementary Figure S3. The view of the simulated AA-stacking (a) and staggered AB-stacking (b) model of SC-COF$_{TAZ}$.

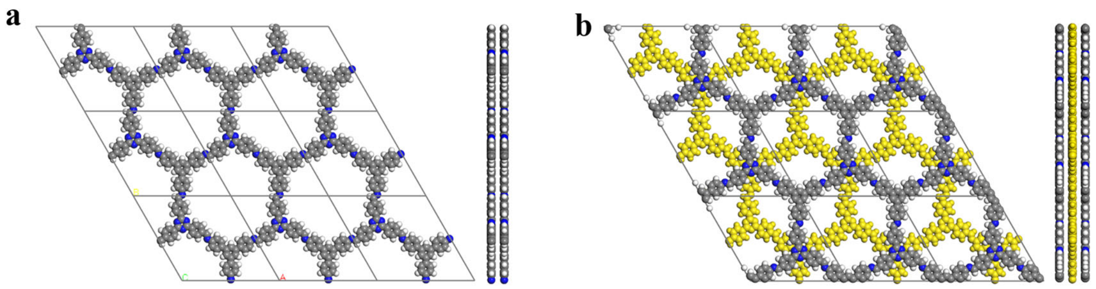

Supplementary Figure S4. The view of the simulated AA-stacking (a) and staggered AB-stacking (b) model of SC-COF$_{Ph}$.

Electrochemical measurements

RRDE measurements:

The 2e^{-} ORR performance of COF and COF@CNT composites was investigated using a rotating ring-disk electrode (RRDE, disk: Φ = 5.61 mm, Pt ring: Φ = 6.25 mm) device (CHI 760E, Shanghai Chenhua Instrumentation Co., Ltd., Shanghai, China). The reaction vessel is a five-necked electrolytic cell with a volume of 250 mL.

Three-electrode system was employed for performance testing, and the calibration of the reference electrode was conducted before the test. A saturated calomel electrode (SCE) served as the reference electrode, a Pt sheet as the counter electrode, and a Pt mesh as the working electrode. The electrolyte used was 0.1 M KOH solution, and hydrogen gas was bubbled through the electrolyte to saturate the dissolved hydrogen content. Linear sweep voltammetry (LSV) measurements were performed at a scan rate

of 5 mV s^{-1}, and the potential at the zero-current point was defined as the calibrated potential.

The preparation procedure of the electrolyte was as follows: 5.61 g (for 0.1 M KOH solution) and 56.1 g (for 1.0 M KOH solution) of KOH were accurately weighed respectively and fully dissolved in 1000 mL of deionized water. After cooling the solution to room temperature, a pH meter was used for calibration to adjust the pH value to 12.97 (±0.3) or 13.95 (±0.3).

The apparent collection efficiency (*N*) measurements of RRDE were performed in a mixed solution of KOH (0.1 M) and $K_3[Fe(CN)_6]$ (0.01 M) under Ar atmosphere, and detailed procedure was as follows: LSV curves were tested at different rotational speeds over a potential range of 1.5 V to 0.4 V (*vs.* RHE) with a scan rate of 10 mV s^{-1}, and the potential of the ring electrode was set to 1.55 V (*vs.* RHE). The *N* was obtained by fitting the linear relationship between the disk current and the ring current at different rotational speeds.

Catalyst performance testing: RRDE was polished and cleaned using Al_2O_3 (50 nm), after which catalyst powders containing different COF materials were uniformly drop-coated on the disk electrodes with a catalyst loading of 0.1 mg cm^{-2}. Catalyst ink preparation: 3.3 mg of catalyst powder was accurately weighed and dispersed in 980 μL of isopropanol and sonicated for After 30 minutes, 20 μL of nafion (5 wt%) solution was added and continued sonication for 60 minutes to obtain the catalyst ink. Three-electrode test system was constructed with a carbon rod as the counter electrode, a saturated calomel electrode (SCE, 0.2415 V) as the reference electrode, and a catalyst modified glassy carbon as the working electrode. The potential was converted to RHE according to the equation:

$$E(vs.RHE) = E_0(vs.SCE) + 0.2415 + 0.059 \times pH \tag{1}$$

The cyclic voltametric (CV) scan was first performed for 50 turns in Ar-saturated 0.1 M KOH solution for activation and stabilization of the catalyst, with a scan rate of 50 mV s^{-1} and a potential ranging from 1.0 V to 0 V (*vs.* RHE). After that, the potential of the Pt-ring electrode was set to 1.2 V (*vs.* RHE), and the LSV curves of the disk electrode were scanned in Ar- and O_2-saturated 0.1 M KOH solutions, respectively, at a scan rate of 10 mV s^{-1} in a potential range of 1.0 V to 0 V (*vs.* RHE) at a speed of 1600 rpm, with an *i*R compensation of 85%. The actual current was obtained by

subtracting the polarization current of the argon atmosphere, and the H_2O_2 selectivity and electron transfer number (*n*) were calculated by the following equations:

$$H_2O_2(\%) = 200 \times \frac{\frac{i_{Ring}}{N}}{|i_{Disk}| + \frac{i_{Ring}}{N}} \quad (2)$$

$$n = 4 \times \frac{|i_{Disk}|}{|i_{Disk}| + \frac{i_{Ring}}{N}} \quad (3)$$

where i_{Ring} is the ring current, i_{Disk} is the disk current, and *N* is the determined collection efficiency.

Electrochemical impedance spectroscopy (EIS) measurements:

The EIS tests were performed with the disk electrode of the RRDE as the working electrode at a potential of 0.7 V (*vs.* RHE), with scanning frequencies from 100 kHz to 0.1 Hz.

Electrochemcial active surface area (ECSA) measurements:

The ECSA of molecular catalysts was evaluated using C_{dl}. The CV curves of the molecular catalysts were measure at different scan rates (10, 20, 30, 40, and 50 mV s^{-1}) over the potential range of 0.8085 V to 0.9085 V (*vs.* RHE) using the disk electrode of RRDE as the working electrode. The electrolyte was an O_2-saturated 0.1 M KOH aqueous solution. C_{dl} was estimated by plotting (j_a-j_b)/2 versus scan rate at 0.8585 V, where j_a and j_b are the anodic and cathodic current densities, respectively.

TOF analysis: The TOF values of the COF materials were calculated using the following equation:

$$TOF = \frac{(i_{H_2O_2} \times M)}{2 \times F \times m \times \omega} \quad (4)$$

$$i_{H_2O_2} = \frac{i_{Ring}}{N} \quad (5)$$

Where: i_{Ring} is the ring current; *M* is the molar mass of the COF material (smallest structural unit); *F* is the Faraday constant (96485 C mol^{-1}); *m* is the catalyst loading; and *ω* is the mass fraction of COF in the catalyst.

The entire ORR reaction pathway in alkaline environment is:

The 2e⁻ ORR:

$$O_2 + H_2O + 2e^- \rightarrow HO_2^- + OH^- \; E^{\theta} = 0.7\,V\,vs.RHE \quad (6)$$

Which can be divided into steps as follows:

$$* + O_2 + H_2O + e^- \rightarrow * OOH + OH^- \quad (7)$$

$$* OOH + e^- \rightarrow * + HO_2^- \quad (8)$$

The 4e⁻ ORR:

$$O_2 + 2H_2O + 4e^- \rightarrow 4OH^- \; E^{\theta} = 1.23\,V\,vs.RHE \quad (9)$$

Which can be divided into steps as follows:

$$* + O_2 + H_2O + e^- \rightarrow * OOH + OH^- \quad (10)$$

$$* OOH + e^- \rightarrow * O + OH^- \quad (11)$$

$$* O + H_2O + e^- \rightarrow * OH + OH^- \quad (12)$$

$$* OH + e^- \rightarrow * + OH^- \quad (13)$$

For each step, * represents the catalytic active site.

The adsorption energy and reaction Gibbs free energy calculations

The adsorption energy (E_{ads}) is calculated by using the following equation:

$$E_{ads} = E_{COF/OOH} - E_{COF} - E_{OOH} \quad (14)$$

Where $E_{COF/OOH}$ represents the energy of COF after adsorption of OOH, E_{COF} represents the energy of COF, and E_{OOH} represents the energy of OOH.

We employed the computational hydrogen electrode (CHE) model to calculate the Gibbs free energy change (ΔG) of the proton-coupled electron transfer (PCET) step. The CHE model uses one-half of the chemical potential of hydrogen as the chemical potential of the proton-electron pair. The ΔG value was determined using the following equation:

$$\Delta G = \Delta E + \Delta ZPE - T\Delta S \quad (15)$$

Where ΔE represents the electronic energy difference between the initial and final states of the PCET step. ΔZPE and TΔS are the zero-point energy difference and the entropy change between the initial and final states, where T is the absolute temperature.

The theoretical overpotential (η) for ORR is determined as:

$$\eta = U_{equilibrium} - U_{limiting} \tag{16}$$

Where $U_{equilibrium}$ is the equilibrium potential of ORR and $U_{limiting}$ is the applied potential for removing the energy barrier of the rate-limiting step.

DFT calculations mainly provide analysis at the theoretical level and need to be combined with experimental results to form a more comprehensive discussion.

2. Supplementary figures and tables

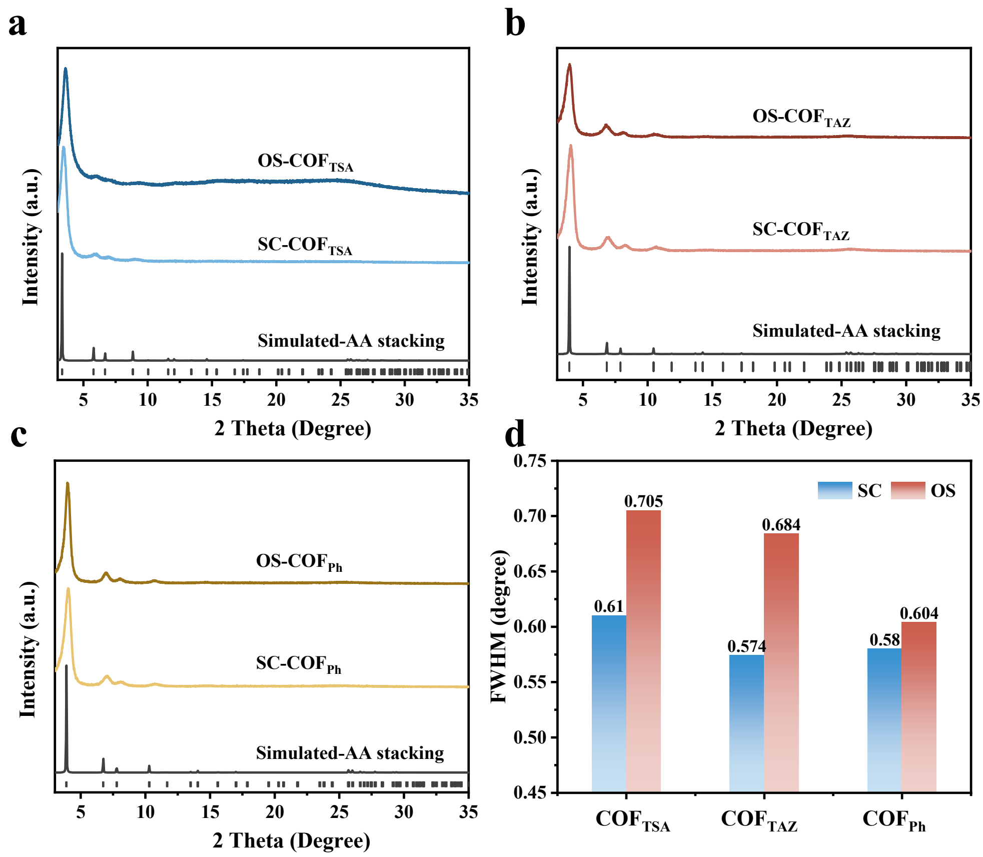

Supplementary Figure 5. Comparison of crystallinity for COFs synthesized by different methods. (a-c) XRD patterns of COFTSA, COFTAZ, and COFPh synthesized by supercritical solvothermal and organic solvothermal method. (d) FWHM values of (100) crystal plane peak for the as-synthesized COFs.

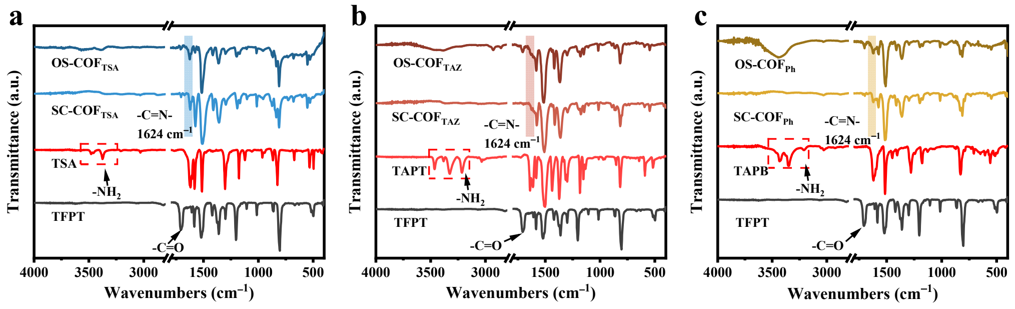

Supplementary Figure 6. FT-IR spectra of COFs synthesized by supercritical solvothermal and organic solvothermal.

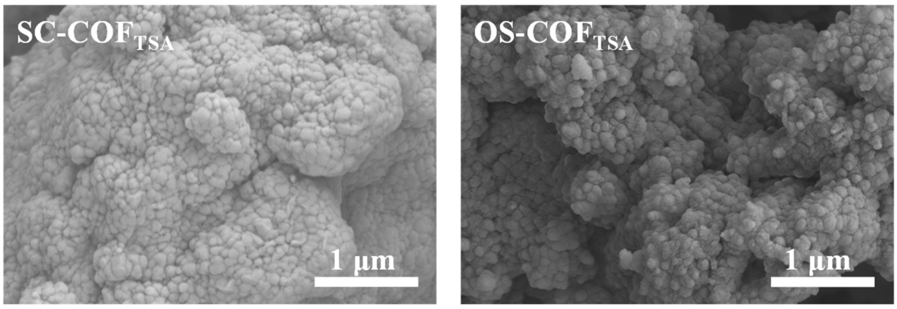

Supplementary Figure 7. SEM images of SC-COF$_{TSA}$, and OS-COF$_{TSA}$.

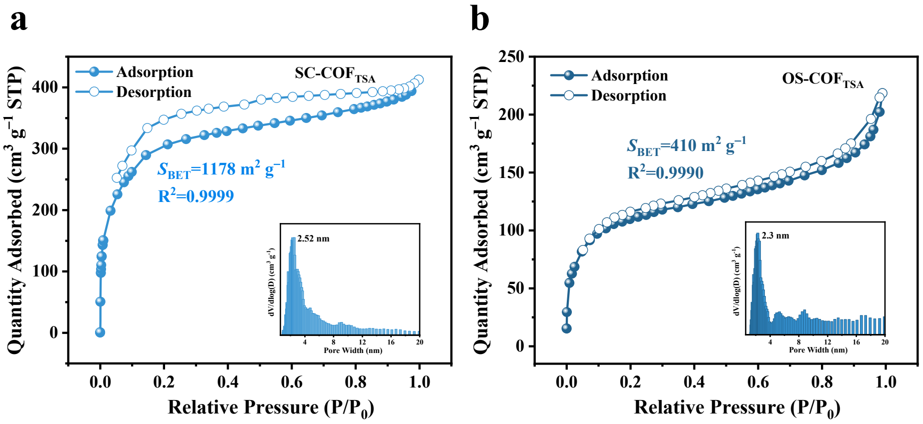

Supplementary Figure 8. N_2 sorption isotherms at 77 K, and Pore size distribution (inset) of SC-COF$_{TSA}$ and OS-COF$_{TSA}$.

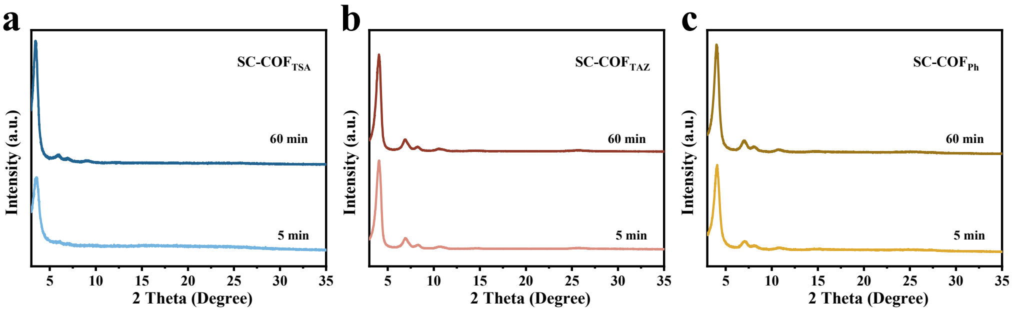

Supplementary Figure 9. Comparison of XRD patterns for SC-COFs synthesized for 5 min and 60 min.

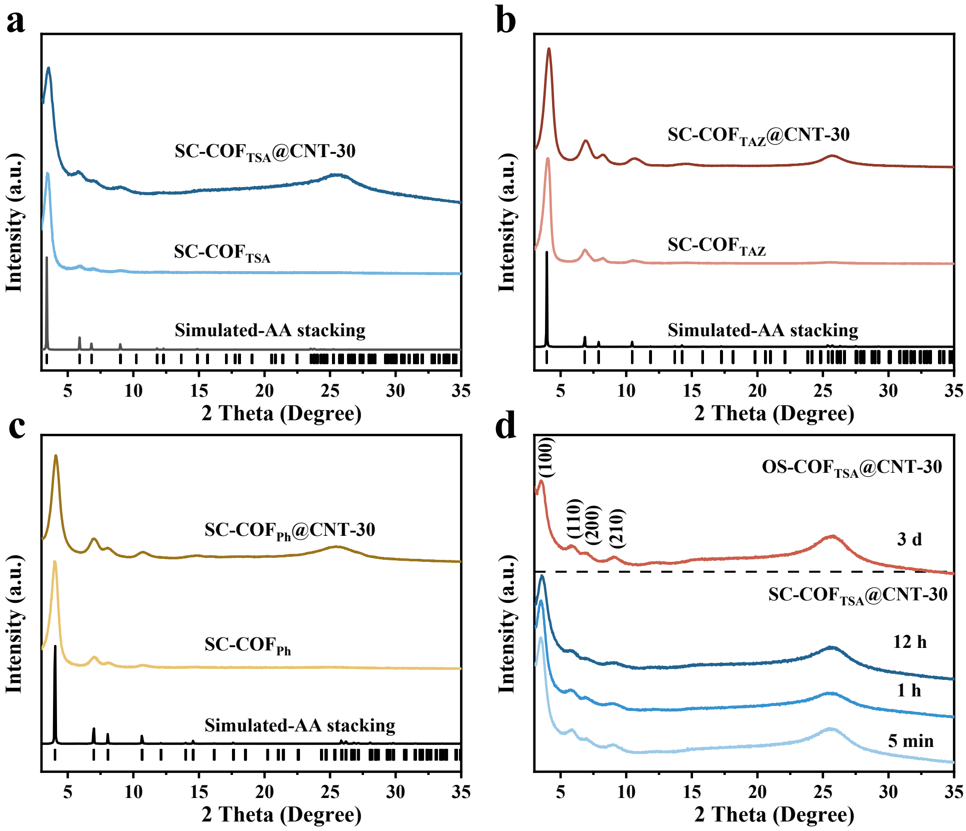

Supplementary Figure 10. XRD characterization of SC-COFs@CNT-30 composites. (a-c) XRD patterns of SC-COFs and SC-COFs@CNT-30 composites. (d) XRD patterns of COFs@CNT-30 synthesized by different methods at different reaction times.

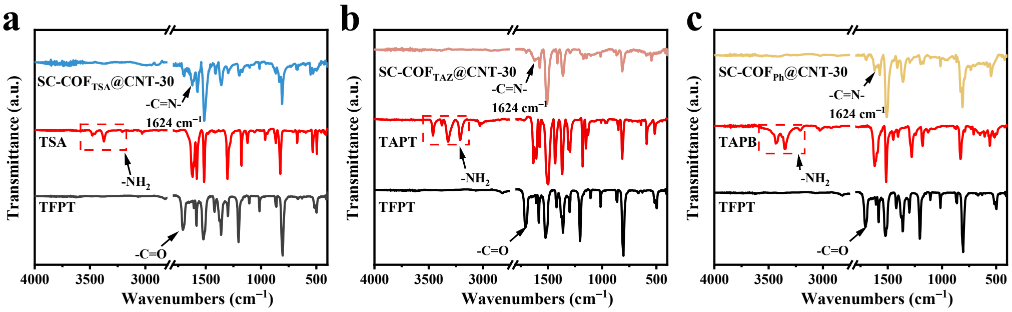

Supplementary Figure 11. FT-IR spectra of (a) SC-COF$_{TSA}$@CNT-30, (b) SC-COF$_{TAZ}$@CNT-30, and (c) SC-COF$_{Ph}$@CNT-30.

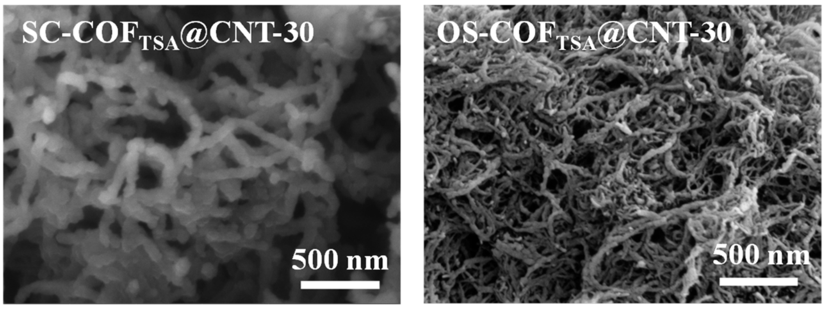

Supplementary Figure 12. SEM images of SC-COF$_{TSA}$@CNT-30 and OS-COF$_{TSA}$@CNT-30.

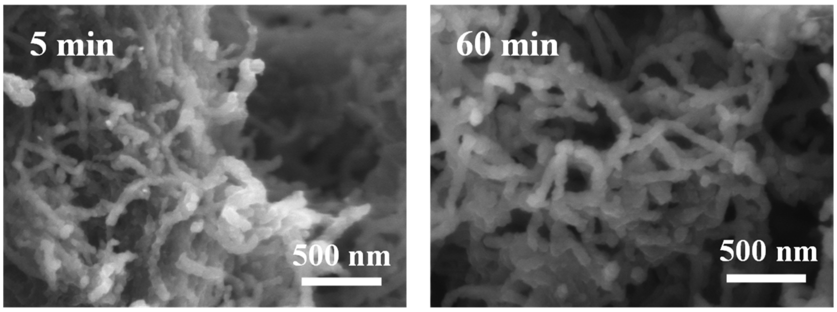

Supplementary Figure 13. SEM images of SC-COF$_{TSA}$@CNT-30 synthesized for 5 min and 60 min.

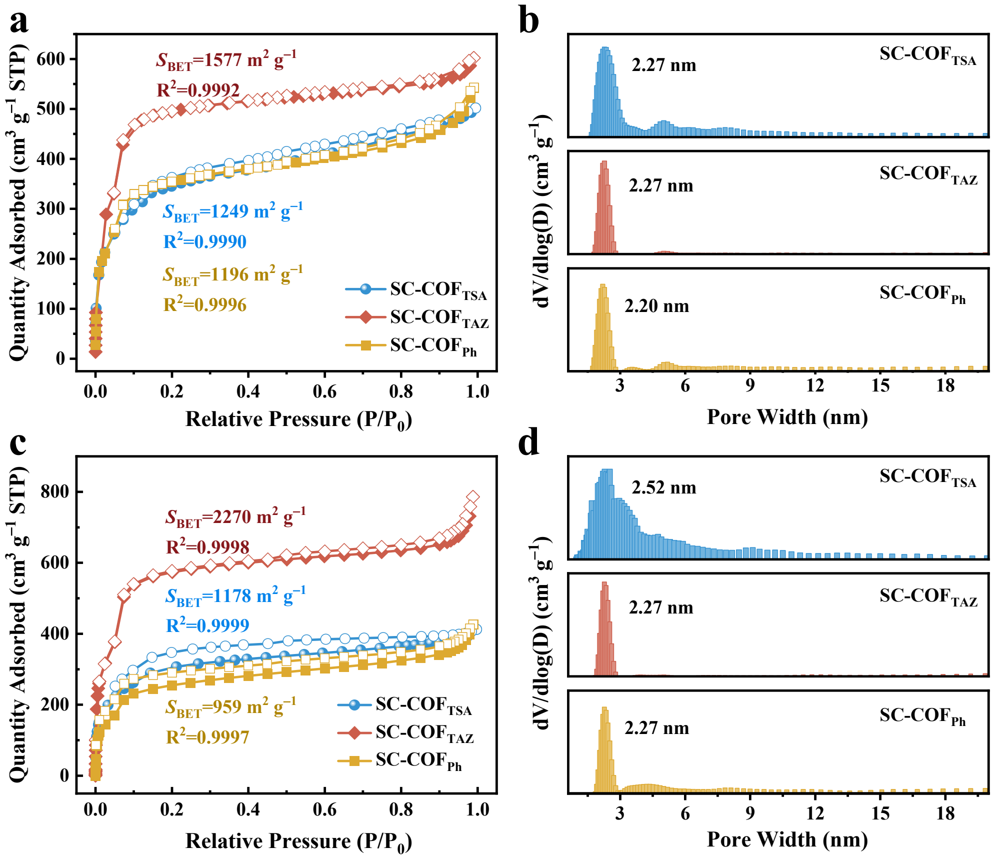

Supplementary Figure 14. N_2 sorption isotherms at 77 K, and Pore size distribution of SC-COFs synthesized for 5 min (a, b) and 60 min (c, d).

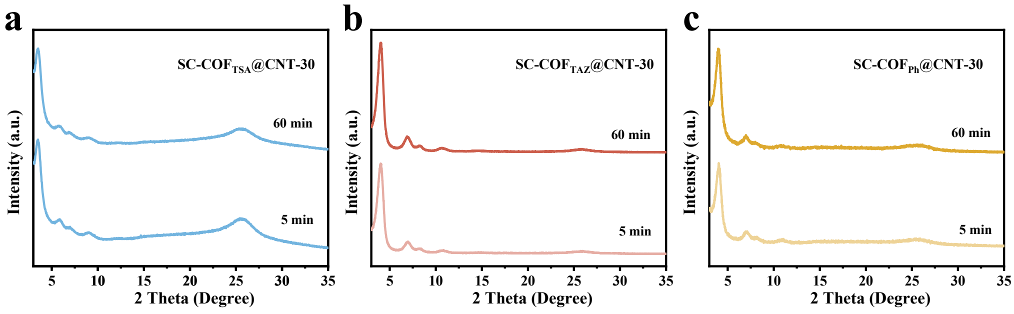

Supplementary Figure 15. Comparison of XRD patterns for SC-COFs@CNT-30 synthesized for 5 min and 60 min.

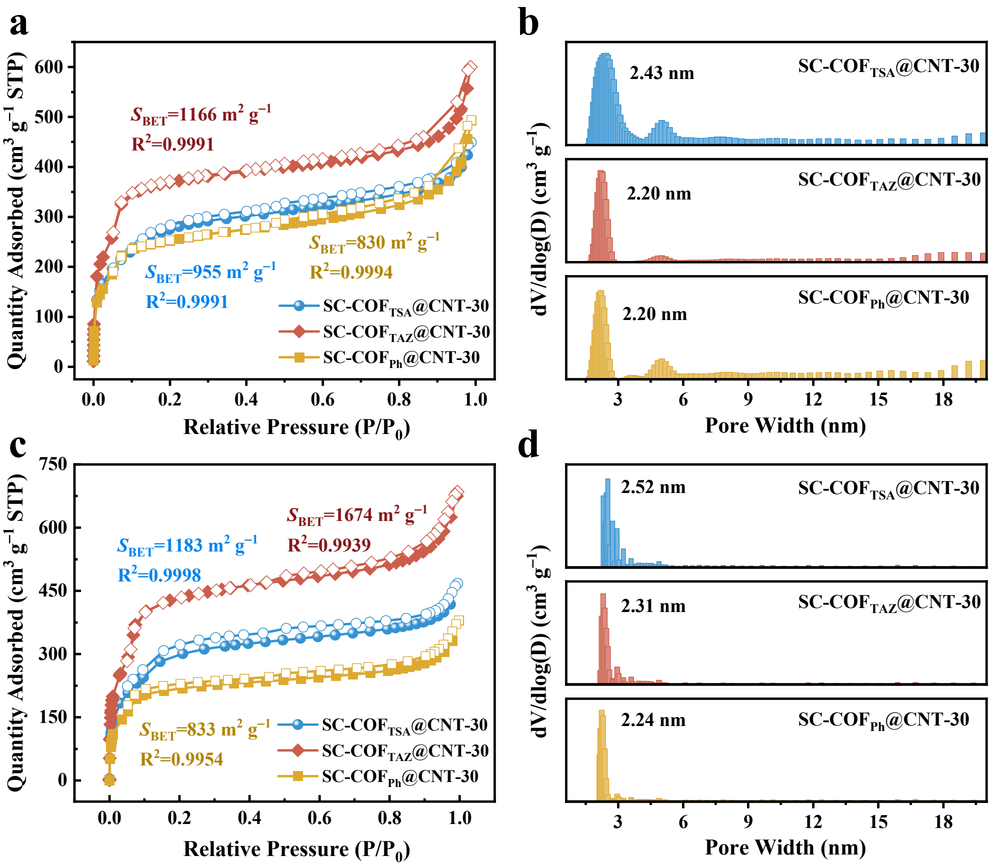

Supplementary Figure 16. N_2 sorption isotherms at 77 K, and Pore size distribution of SC-COFs@CNT-30 synthesized for 5 min (a, b) and 60 min (c, d).

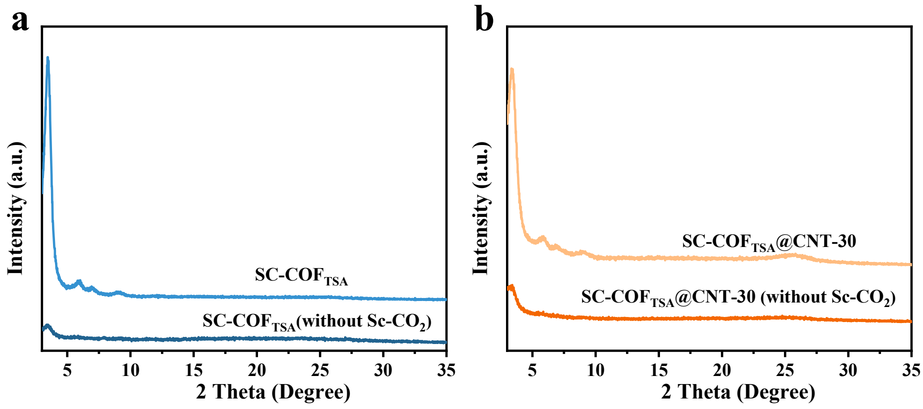

Supplementary Figure 17. Comparison of XRD patterns for SC-COF$_{TSA}$ (a) and SC-COF$_{TSA}$@CNT-30 (b) synthesized with Sc-CO_2 and without Sc-CO_2, illustrating the effect of supercritical CO_2 on crystallinity.

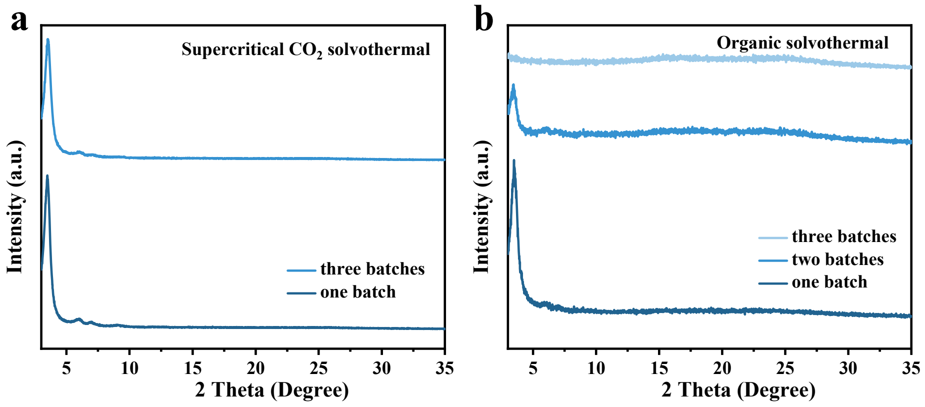

Supplementary Figure 18. XRD patterns of COF_{TSA} synthesized by different methods in various batches.

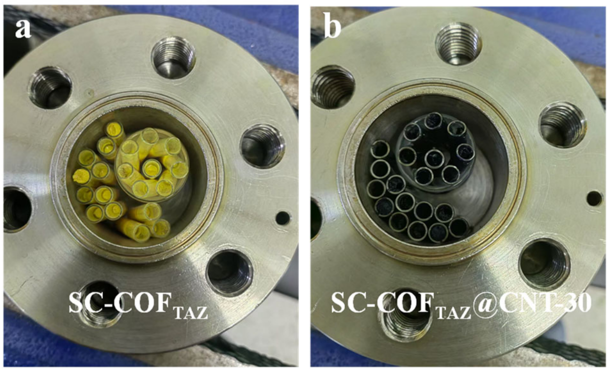

Supplementary Figure 19. Digital images of large-scale-synthesized SC-COF_{TAZ} (a) and SC-COF_{TAZ}@CNT-30 (b) prepared in 20 batches.

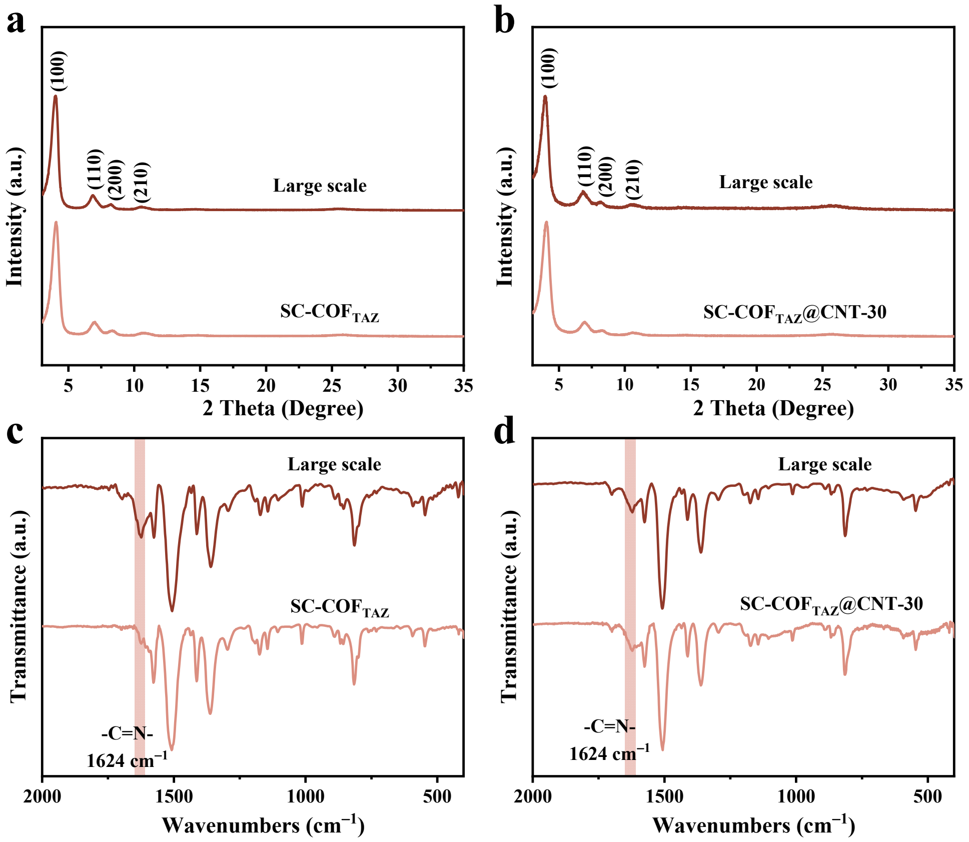

Supplementary Figure 20. XRD patterns (a, b) and FT-IR spectra (c, d) of large-scale-synthesized SC-COF$_{TAZ}$ and SC-COF$_{TAZ}$@CNT-30 prepared in 20 batches.

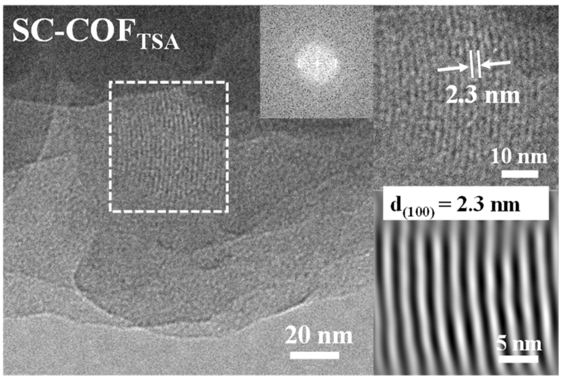

Supplementary Figure 21. HRTEM image of SC-COF$_{TSA}$.

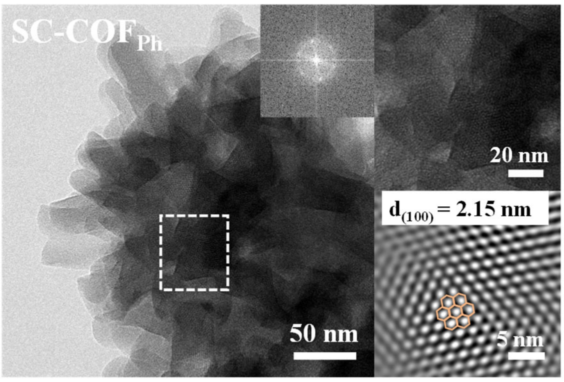

Supplementary Figure 22. HRTEM image of SC-COF$_{Ph}$.

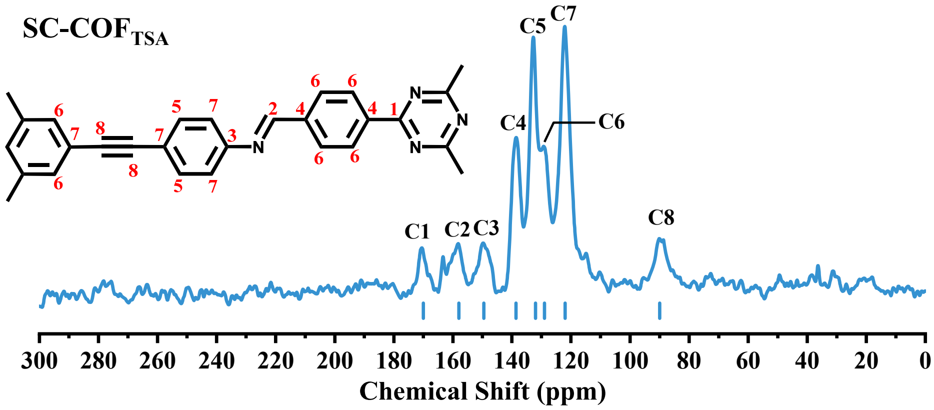

Supplementary Figure 23. Solid-state ^{13}C NMR spectrum of SC-COF$_{TSA}$.

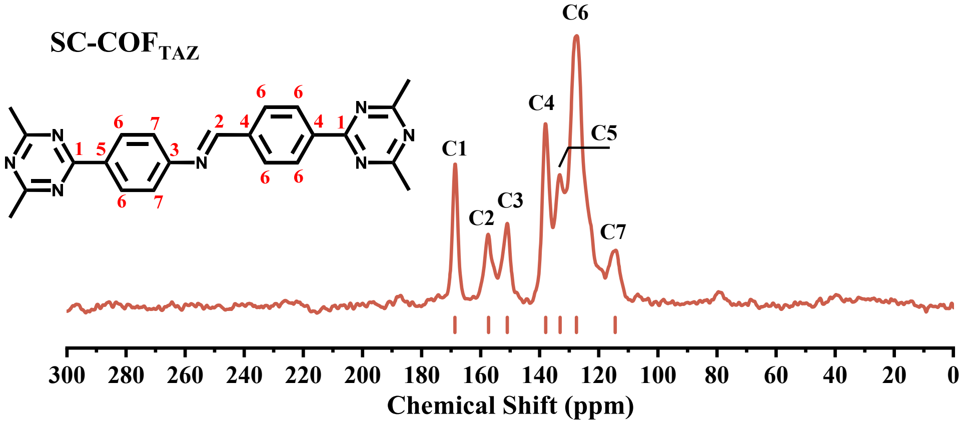

Supplementary Figure 24. Solid-state ^{13}C NMR spectrum of SC-COF$_{TAZ}$.

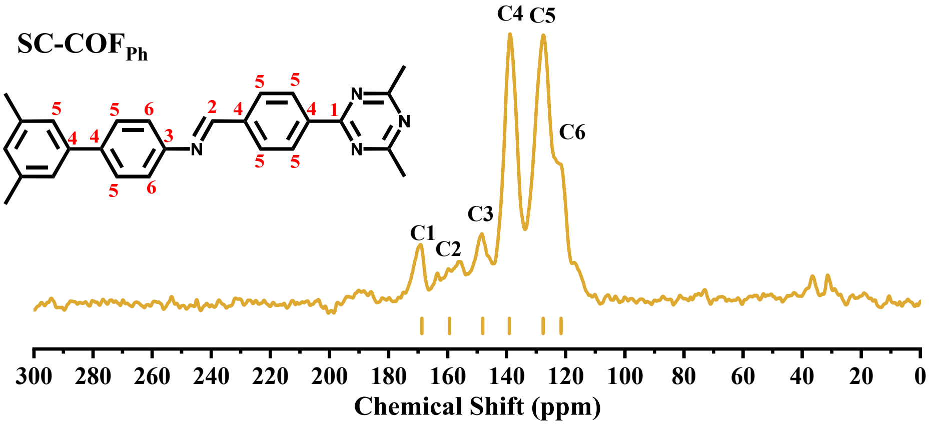

Supplementary Figure 25. Solid-state ^{13}C NMR spectrum of SC-COF$_{Ph}$.

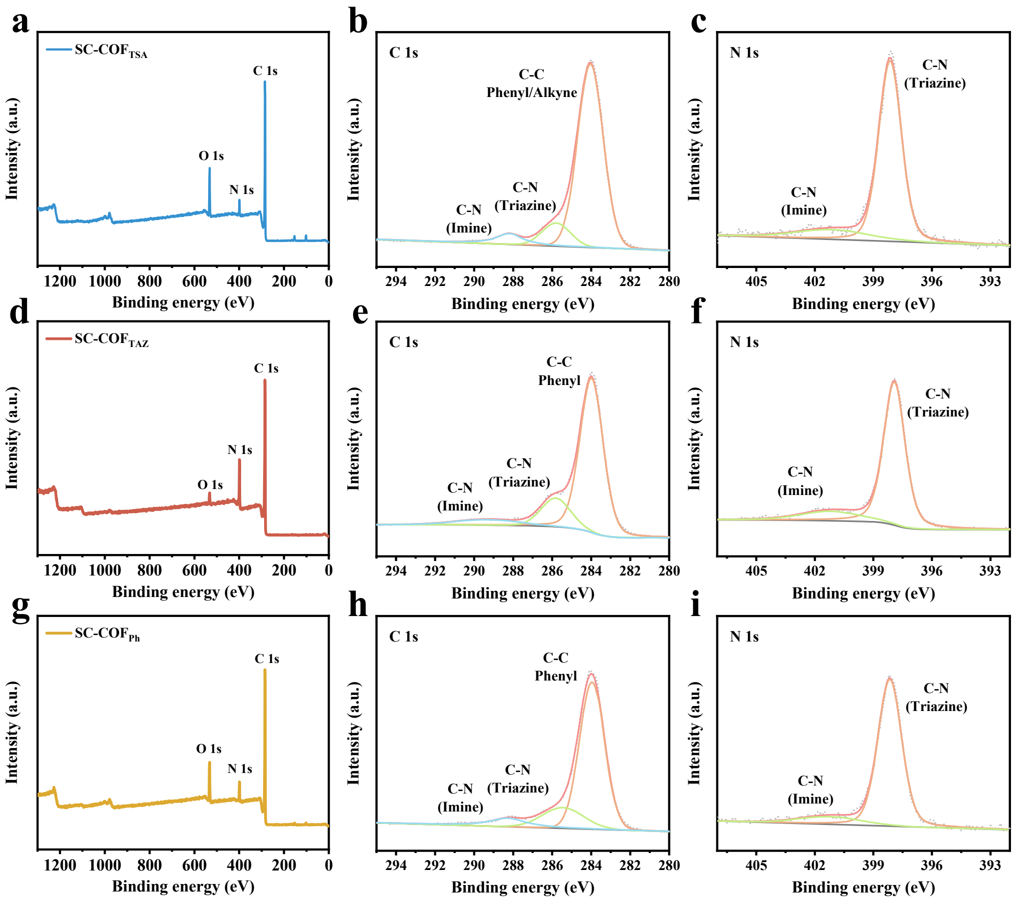

Supplementary Figure 26. XPS spectra of (a-c) SC-COF$_{TSA}$, (d-f) SC-COF$_{TAZ}$, and (g-i) SC-COF$_{Ph}$.

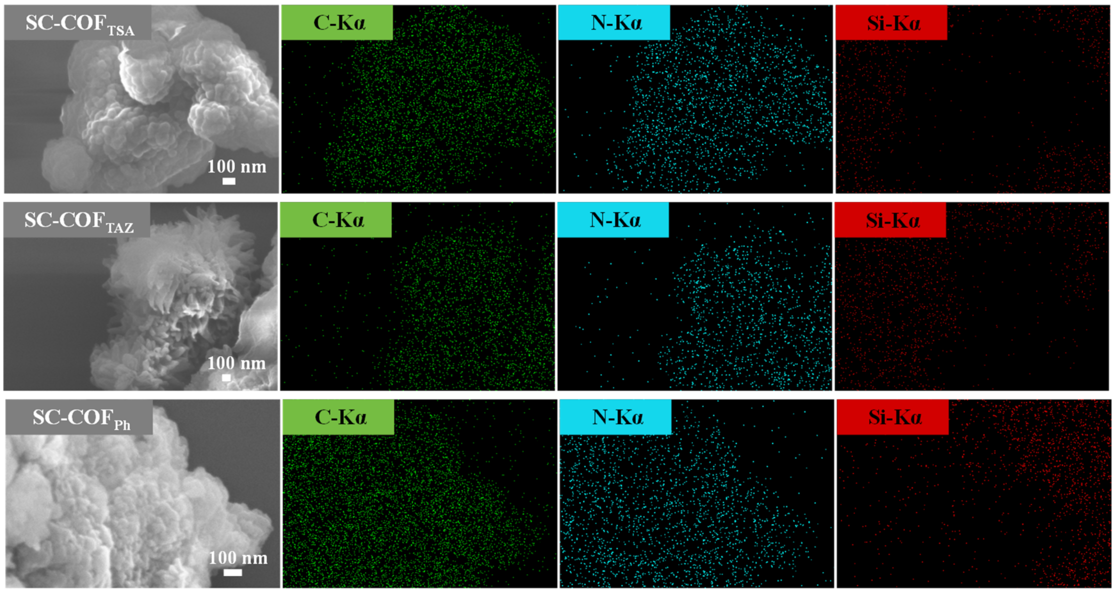

Supplementary Figure 27. EDS mapping images of SC-COF$_{TSA}$, SC-COF$_{TAZ}$, and SC-COF$_{Ph}$.

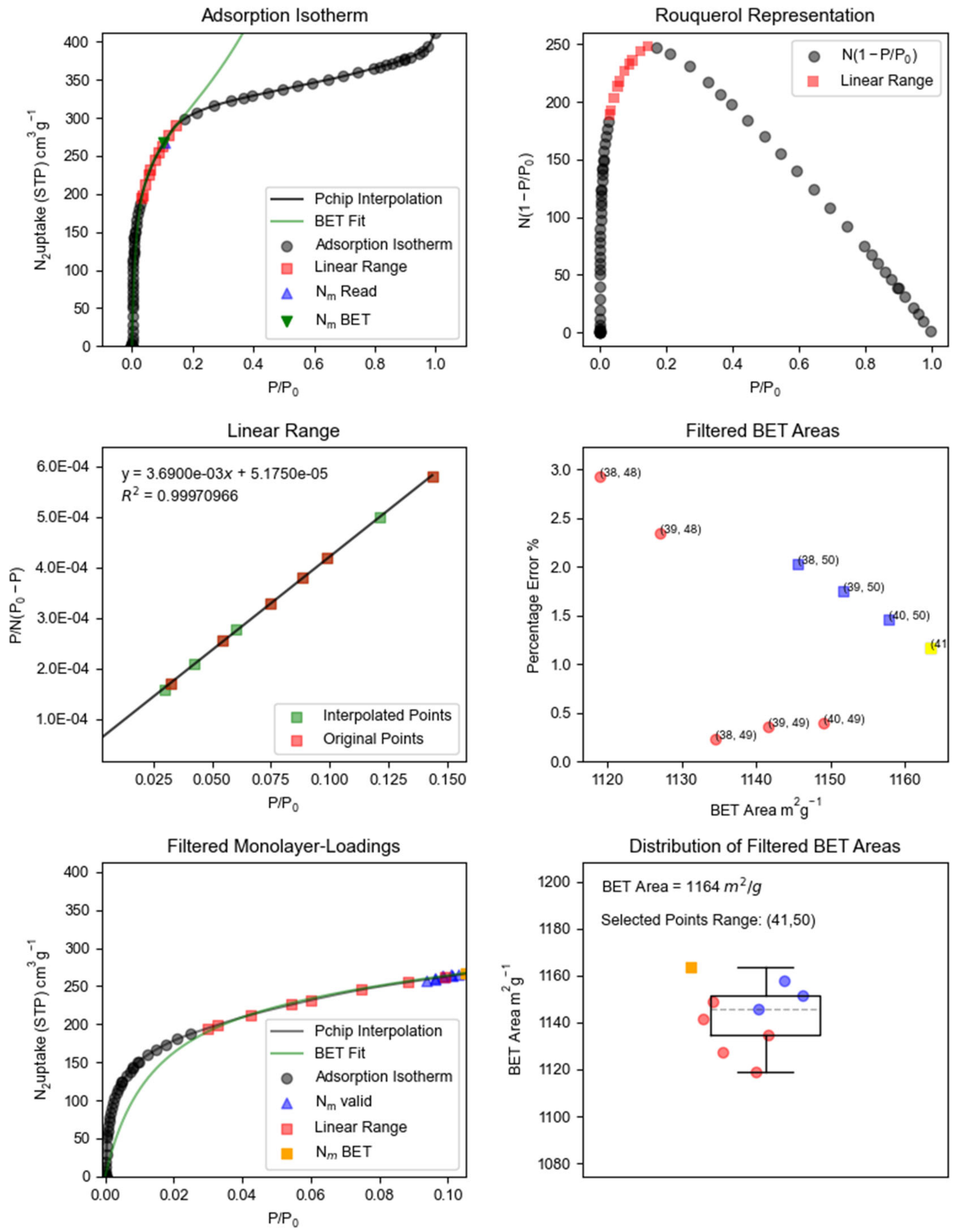

Supplementary Figure 28. BETSI analysis of SC-COF_{TSA}.

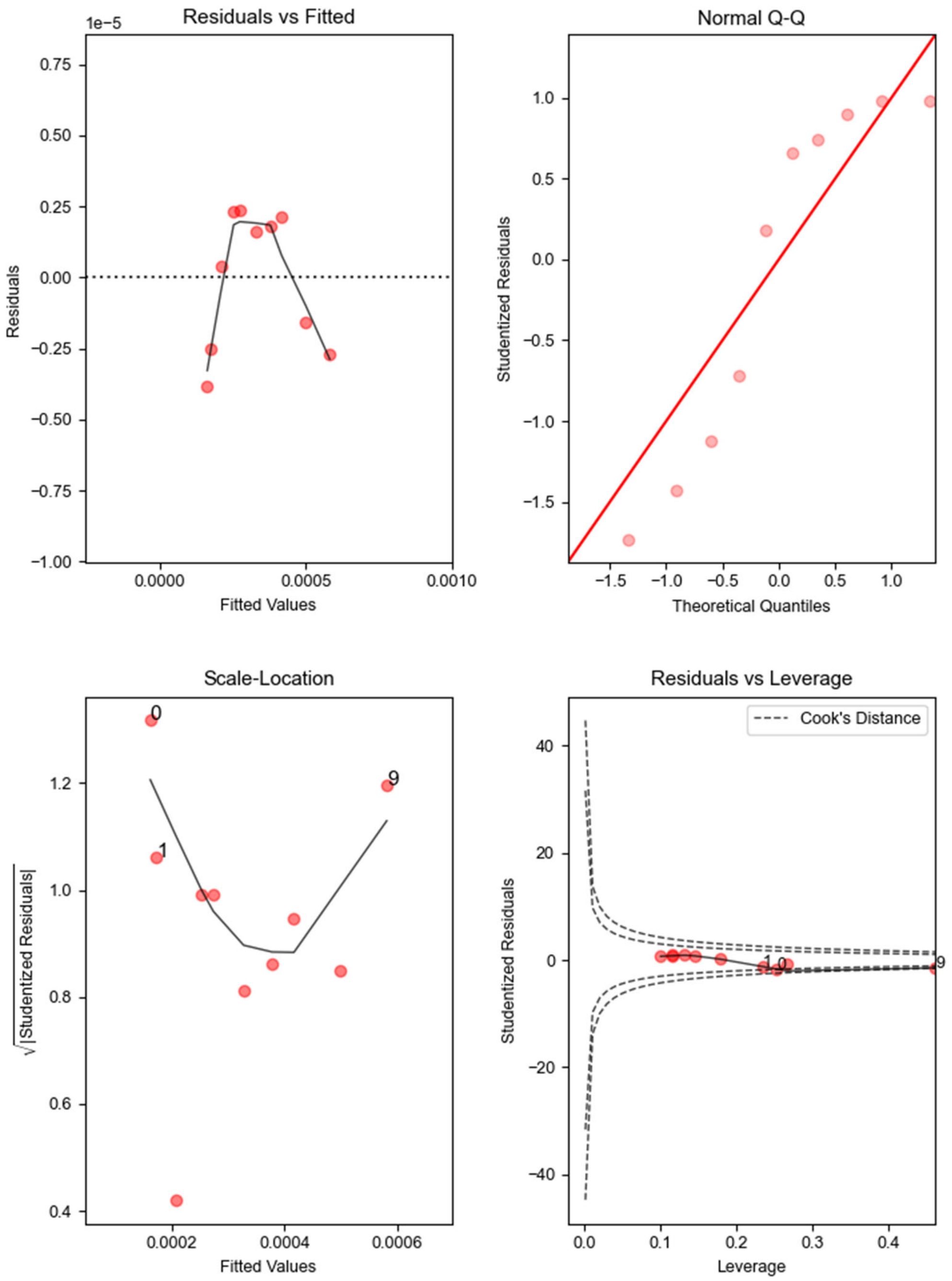

Supplementary Figure 29. BETSI regression diagnostics for SC-COF_{TSA}.

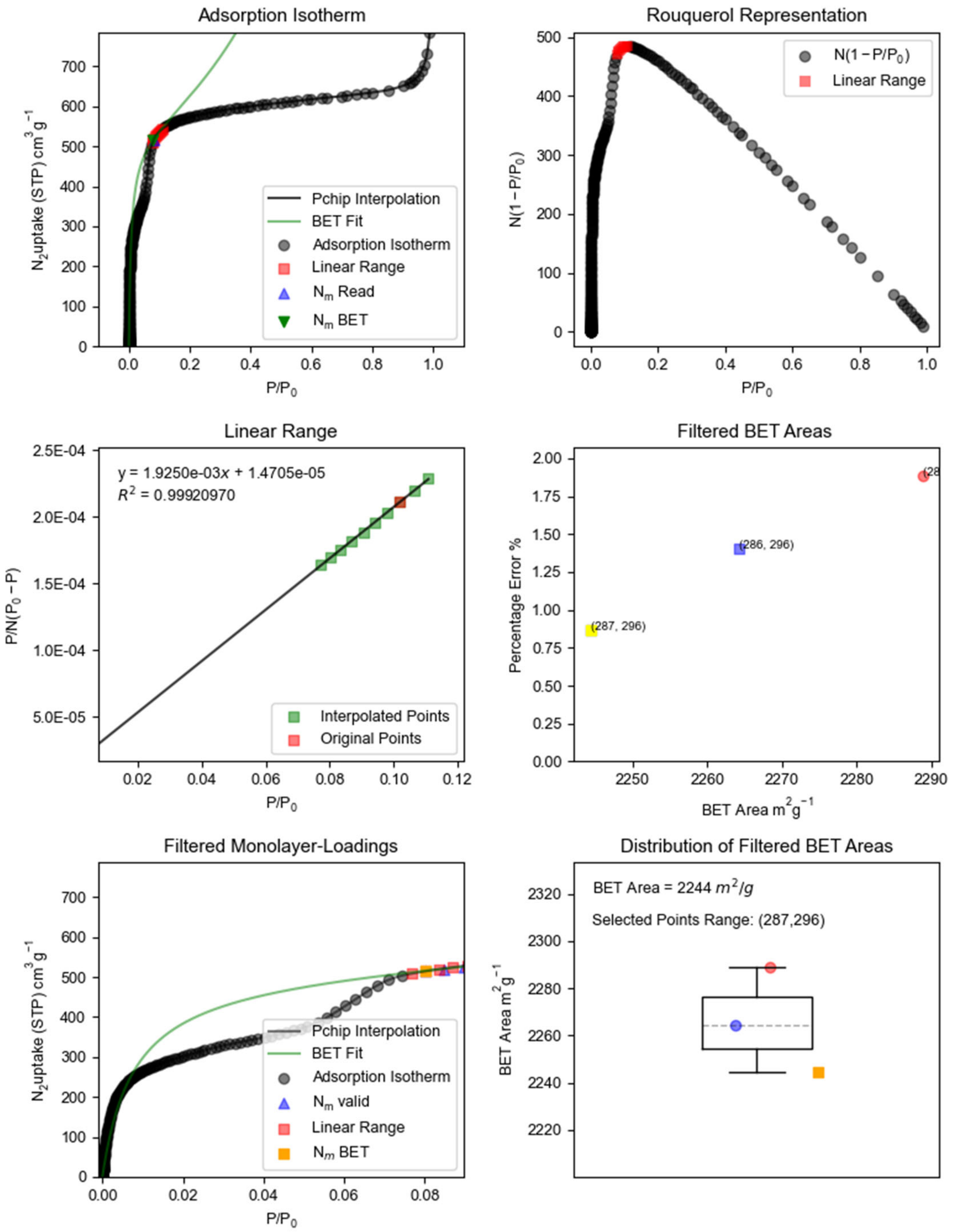

Supplementary Figure 30. BETSI analysis of SC-COF$_{TAZ}$.

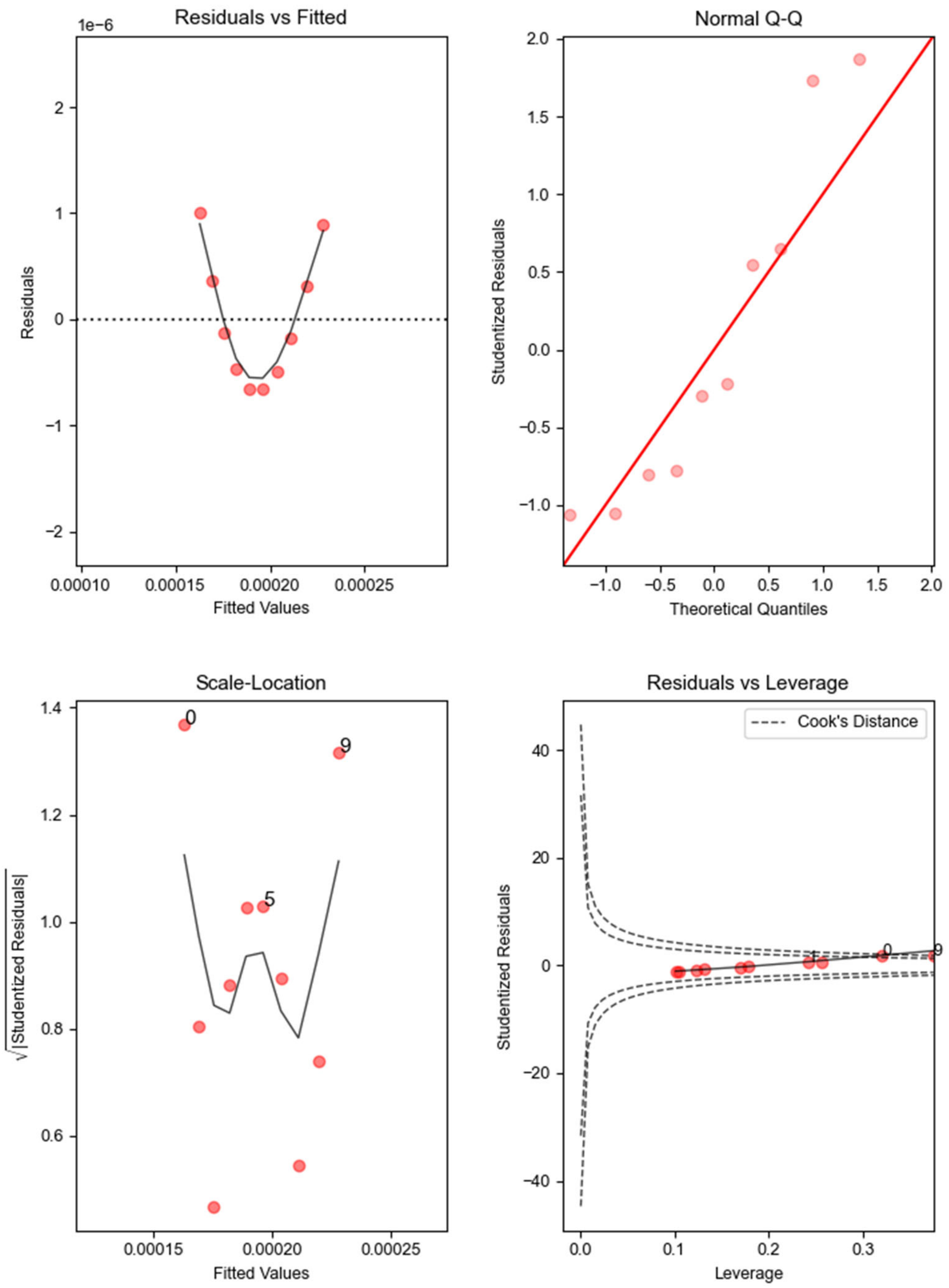

Supplementary Figure 31. BETSI regression diagnostics for SC-COF_{TAZ}.

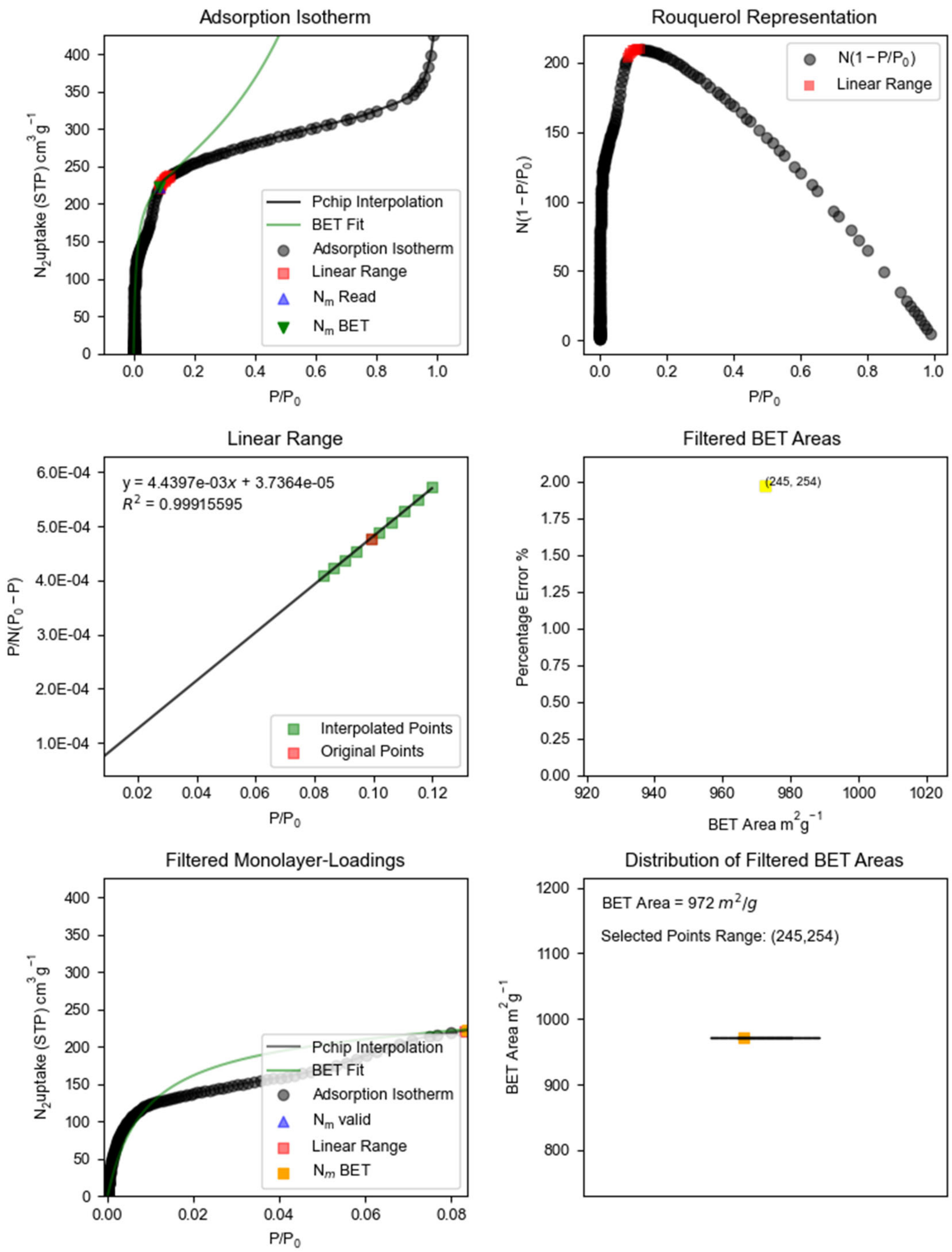

Supplementary Figure 32. BETSI analysis of SC-COF$_{Ph}$.

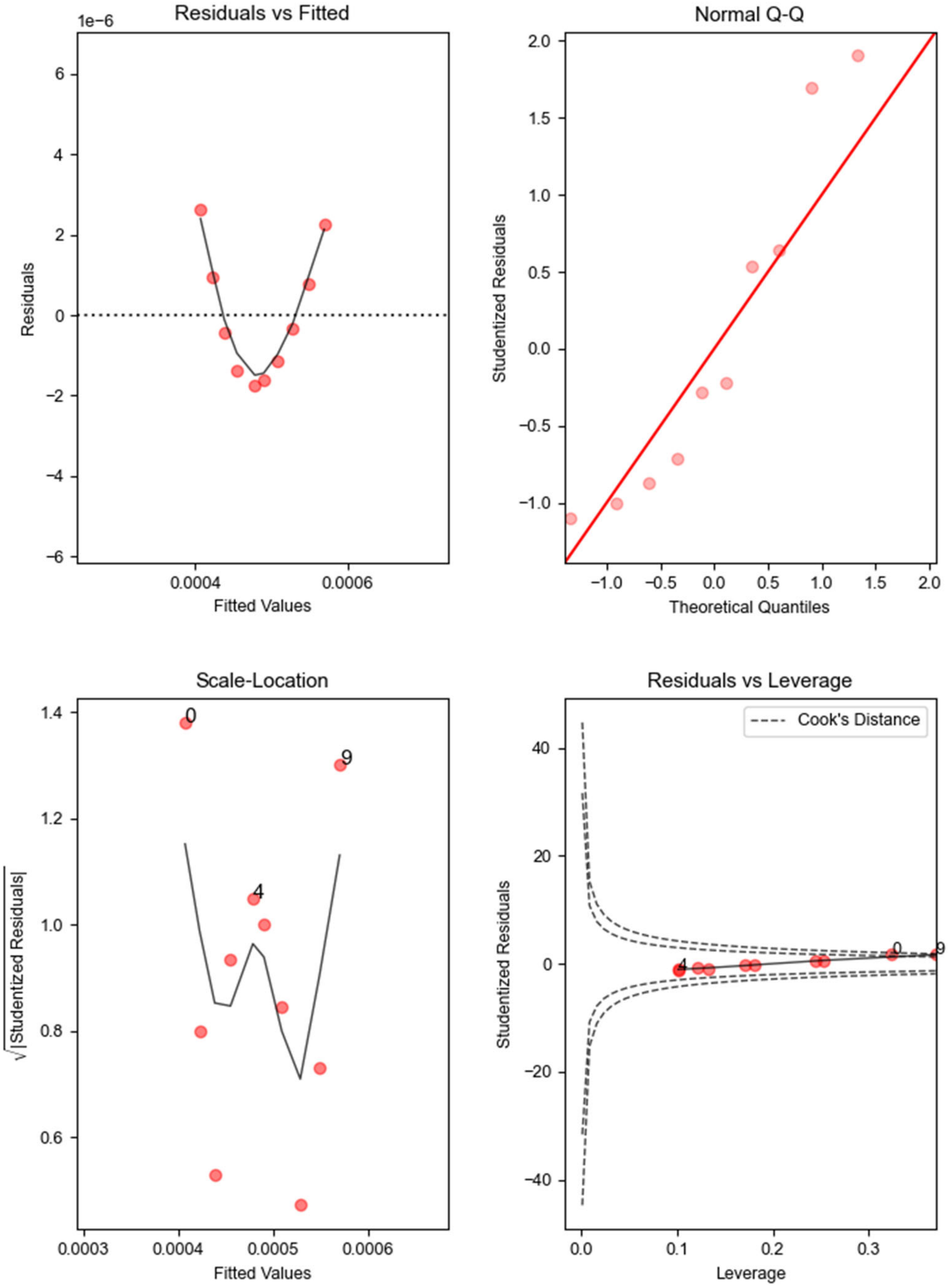

Supplementary Figure 33. BETSI regression diagnostics for SC-COF$_{Ph}$.

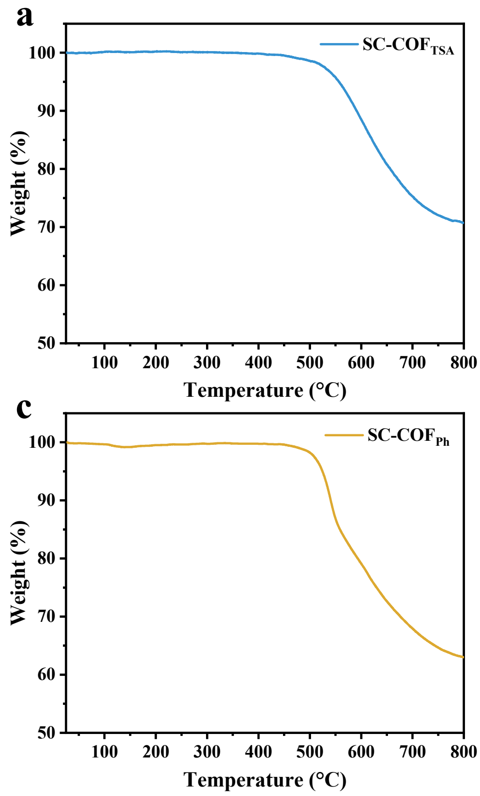

Supplementary Figure 34. TGA trace of (a) SC-COF$_{TSA}$, (b) SC-COF$_{TAZ}$, (c) SC-COF$_{Ph}$, and (d) SC-COF$_{TSA}$ @CNT-30.

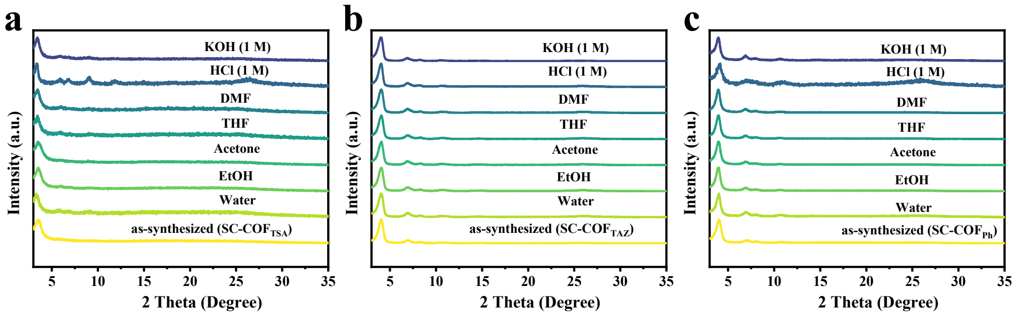

Supplementary Figure 35. XRD patterns of (a) SC-COF$_{TSA}$, (b) SC-COF$_{TAZ}$, and (c) SC-COF$_{Ph}$ after immersion under different solvent conditions for one week.

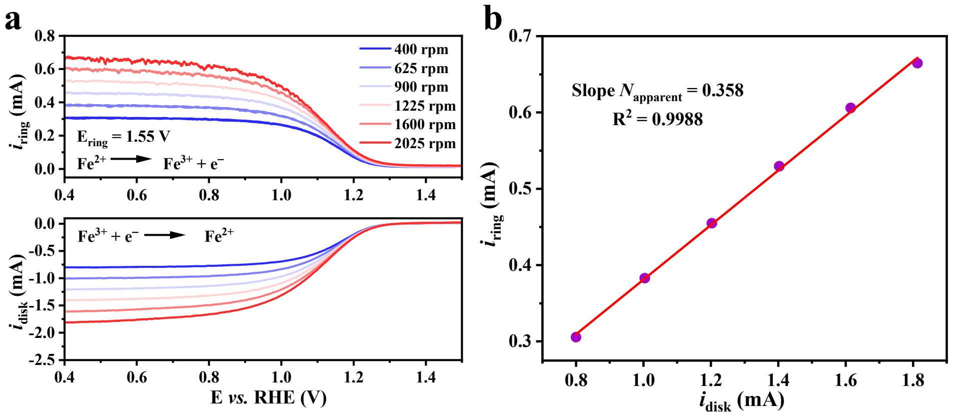

Supplementary Figure 36. RRDE collection efficiency calibration (*N*). (a) Linear sweep voltametric (LSV) curves recorded on a bare glassy carbon rotation disk electrode (Φ = 5.61 mm) with a Pt ring (Φ = 7.91 mm) in the electrolyte of 0.1 M KOH + 10 mM $K_3[Fe(CN)_6]$. Sweep rate: 10 mV s^{-1}, E_{ring} = 1.55 V *vs.* RHE. (b) Linear fitting of the diffusion limited current densities recorded on ring and disk electrodes at different rotation speed. The experimental determined *N* is 35.8%, close to the theoretical vale of 37.0% (Pine AFE7R9).

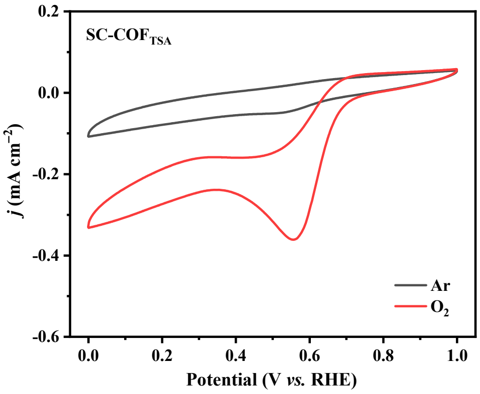

Supplementary Figure 37. CV test of SC-COF_{TSA} in 0.1 M KOH solution saturated with Ar or O_2.

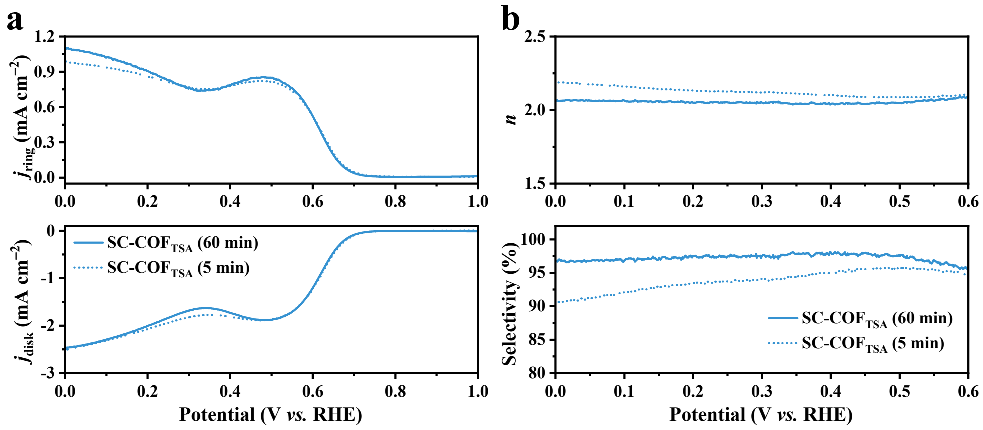

Supplementary Figure 38. The ORR performance comparison of SC-COF$_{TSA}$ synthesized for 5 min and 60 min.

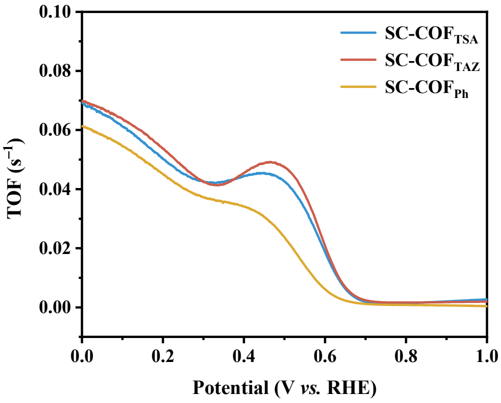

Supplementary Figure 39. The TOF of SC-COF$_{TSA}$, SC-COF$_{TAZ}$, and SC-COF$_{Ph}$.

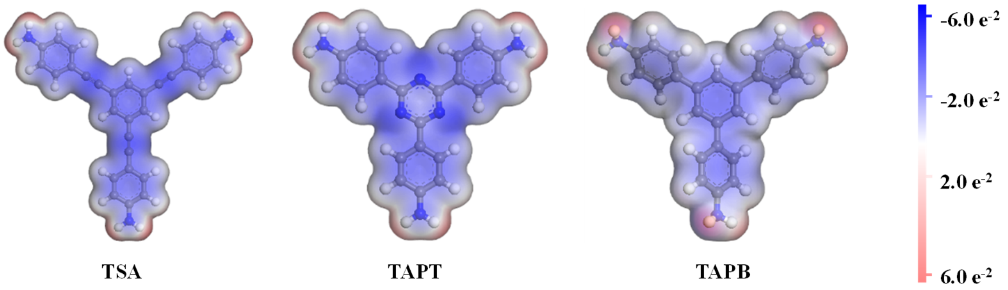

Supplementary Figure 40. ESP analysis of monomers TSA, TAPT, and TAPB.

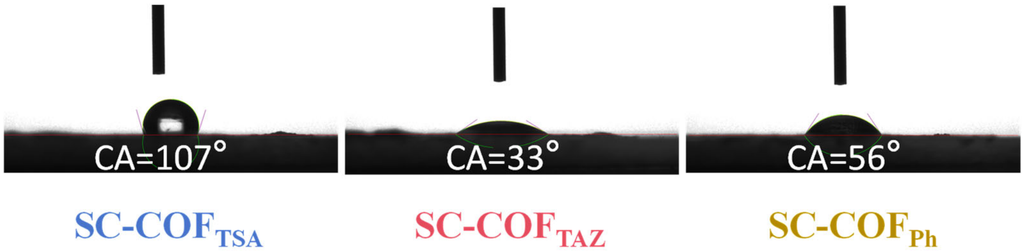

Supplementary Figure 41. Contact angles of SC-COF$_{TSA}$, SC-COF$_{TAZ}$, and SC-COF$_{Ph}$.

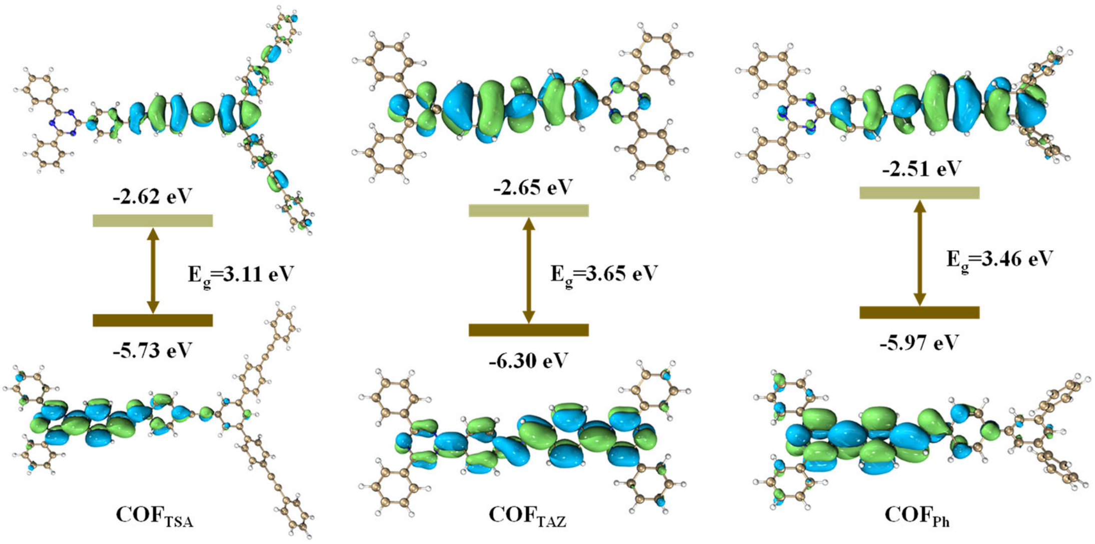

Supplementary Figure 42. LUMO-HOMO models of COF$_{TSA}$, COF$_{TAZ}$, and COF$_{Ph}$.

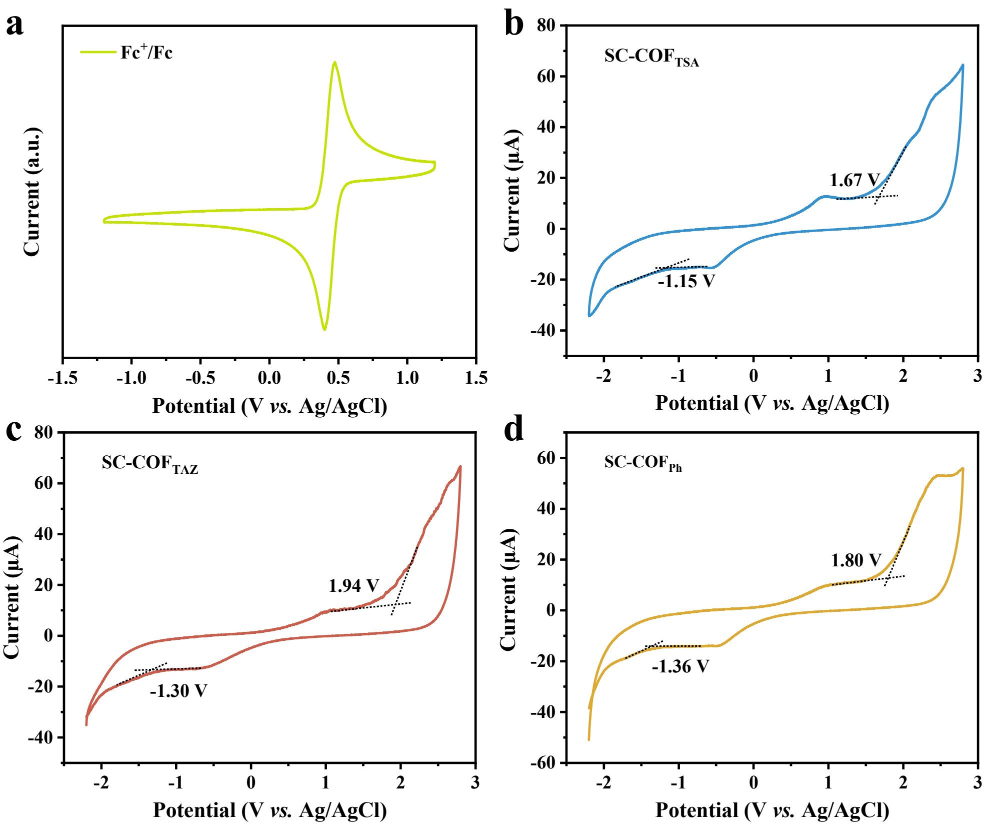

	φ_{ox} (V *vs.* Ag/AgCl)	φ_{red} (V *vs.* Ag/AgCl)	E_{HOMO} (eV)	E_{LUMO} (eV)	$E_{(LUMO-HOMO)}$ (eV)
Fc^+/Fc	0.48	0.40	-	-	-
SC-COF$_{TSA}$	1.67	-1.15	-6.03	-3.21	2.82
SC-COF$_{TAZ}$	1.94	-1.30	-6.3	-3.06	3.24
SC-COF$_{Ph}$	1.80	-1.30	-6.16	-3.00	3.16

Highest occupied molecular orbital (HOMO) and lowest unoccupied molecular orbital (LUMO) energy levels were evaluated via CV using COF-loaded glassy carbon electrodes (0.2 mg cm^{-2}, Φ = 3 mm) in 0.1 M Bu_4NBF_4/acetonitrile, with Ag/AgCl and Pt wire as reference and counter electrodes, respectively. The reduction onset potential (φ_{red}) of the LUMO energy was determined at 100 mV s^{-1}, while the HOMO energy was calculated from the oxidation onset (φ_{ox}) using the formula:

$$E_{HOMO} = -e\left(\varphi_{ox} - \varphi\left(\frac{Fc^+}{Fc}\right) + 4.8\right)(eV) \tag{19}$$

$$E_{LUMO} = -e\left(\varphi_{red} - \varphi\left(\frac{Fc^+}{Fc}\right) + 4.8\right)(eV) \tag{20}$$

Note: $\varphi(Fc^+/Fc) = [\varphi_{ox}(Fc^+/Fc) + \varphi_{red}(Fc^+/Fc)]/2 = 0.44\ (V\ vs. Ag/AgCl)$

Supplementary Figure 43. Cyclic voltammograms of ferrocene/ferrocenium (Fc/Fc^+) couple, SC-COF$_{TSA}$, SC-COF$_{TAZ}$, and SC-COF$_{Ph}$.

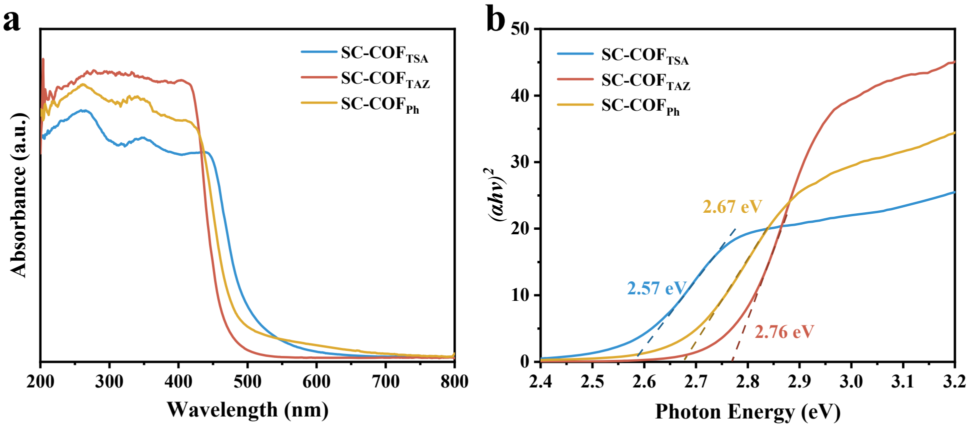

The bandgap energy (E_g) value of the sample could be evaluated using the following equation:

$$(\alpha h\nu)^2 = B(h\nu - E_g) \tag{21}$$

where α is the absorption coefficient, ν is the frequency of the light, h is Planck' s constant, and B is the absorption constant.

Supplementary Figure 44. (a) UV-Vis DRS spectra and (b) Tauc plots of SC-COF$_{TSA}$, SC-COF$_{TAZ}$, and SC-COF$_{Ph}$.

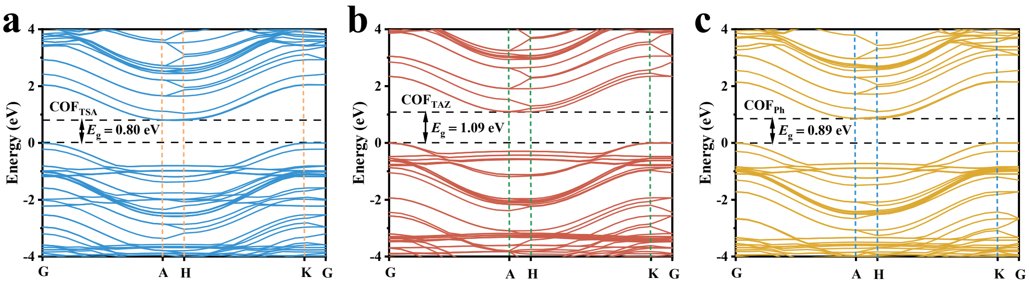

Supplementary Figure 45. Electronic band structures of COF$_{TSA}$, COF$_{TAZ}$, and COF$_{Ph}$.

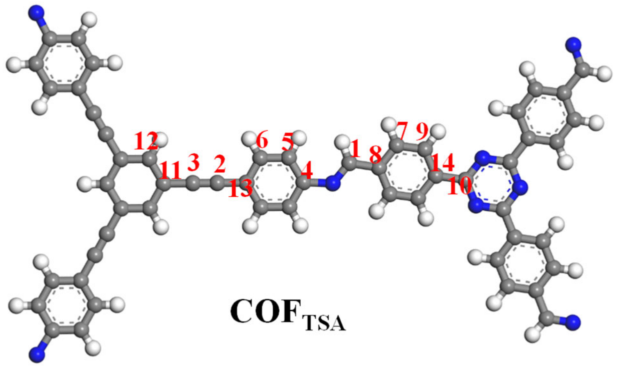

Site	E_{OOH^*} (eV)
1	-3.8213
2	-2.5948
3	-2.7939
4	-2.7220
5	-2.6102
6	-3.0056
7	-2.6762
8	-2.7114
9	-4.5294
10	-2.9366
11	-2.8054
12	-4.1972
13	-2.5948
14	-2.9366

Supplementary Figure 46. The adsorption energy of *OOH at different sites for COF$_{TSA}$ model.

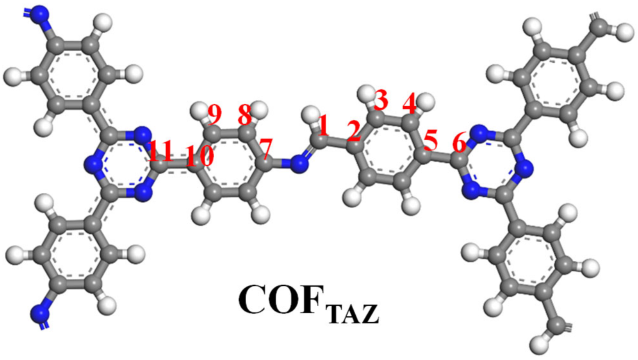

Site	E_{OOH^*} (eV)
**1**	-2.6367
**2**	-2.6737
**3**	-3.2778
**4**	-3.3388
**5**	-3.7104
**6**	-4.4826
**7**	-4.1955
**8**	-3.2786
**9**	-3.1898
**10**	-4.2271
**11**	-4.3939

Supplementary Figure 47. The adsorption energy of *OOH at different sites for COF$_{TAZ}$ model.

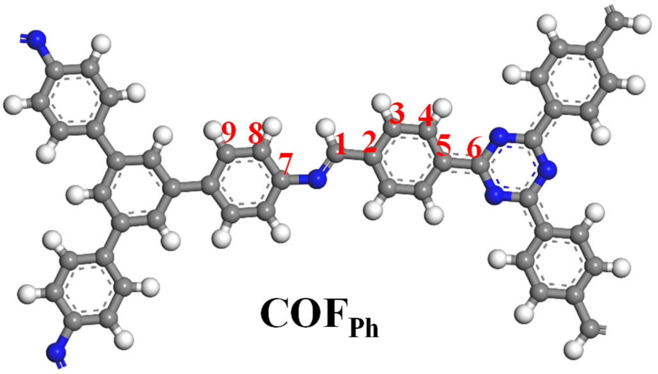

Site	E_{OOH*} (eV)
1	-3.8695
2	-3.7728
3	-3.0319
4	-3.6248
5	-4.4084
6	-4.3408
7	-3.3388
8	-3.2436
9	-5.13163

Supplementary Figure 48. The adsorption energy of *OOH at different sites for COF_{Ph} model.

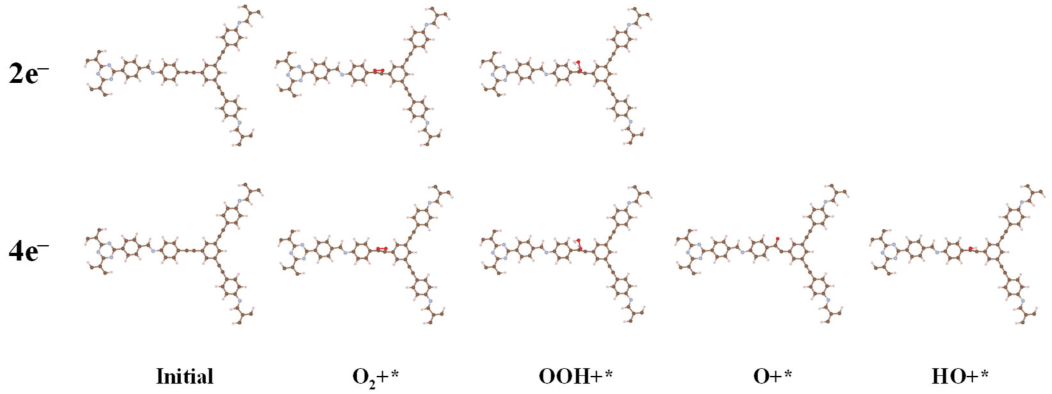

Supplementary Figure 49. The reaction step of O_2 on the site 2 for COF_{TSA} model toward 2e⁻ ORR and 4e⁻ ORR.

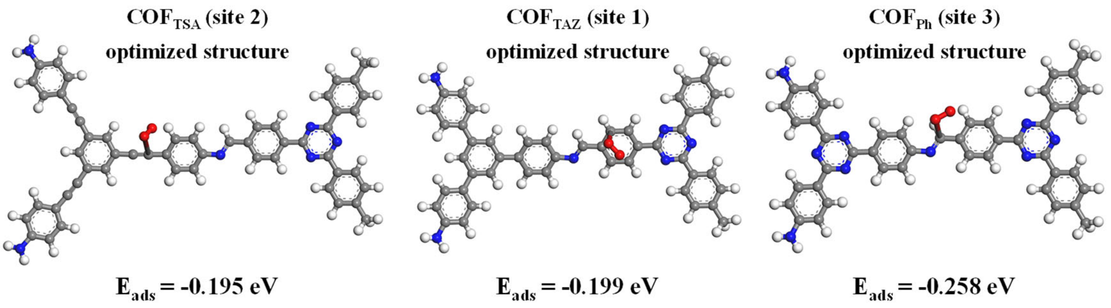

Supplementary Figure 50. The binding energies for COF_{TSA} (site 2), COF_{TAZ} (site 1), and COF_{Ph} (site 3).

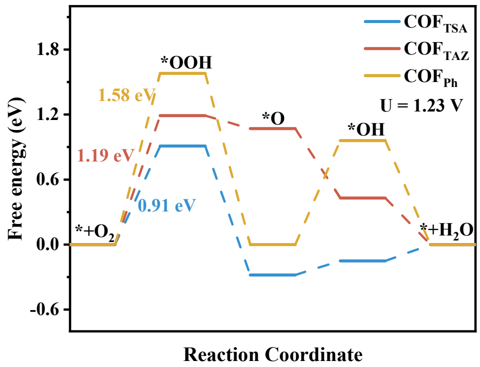

Supplementary Figure 51. Free energy diagrams of 4e⁻ ORR for COF_{TSA} (site 2), COF_{TAZ} (site 1), and COF_{Ph} (site 3).

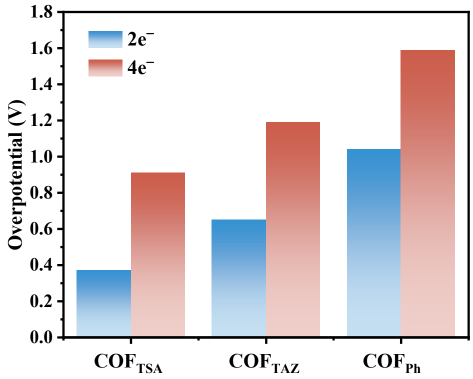

Supplementary Figure 52. The overpotential of COF_{TSA}, COF_{TAZ}, and COF_{Ph} toward $2e^-$ and $4e^-$ ORR.

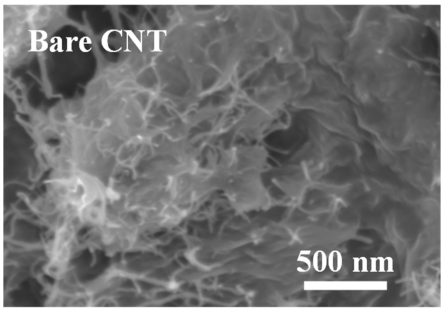

Supplementary Figure 53. SEM image of Bare-CNT.

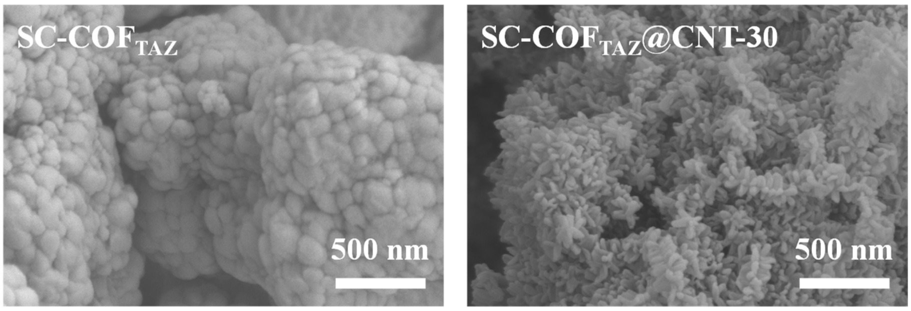

Supplementary Figure 54. SEM images of SC-COF_{TAZ} and SC-COF_{TAZ}@CNT-30.

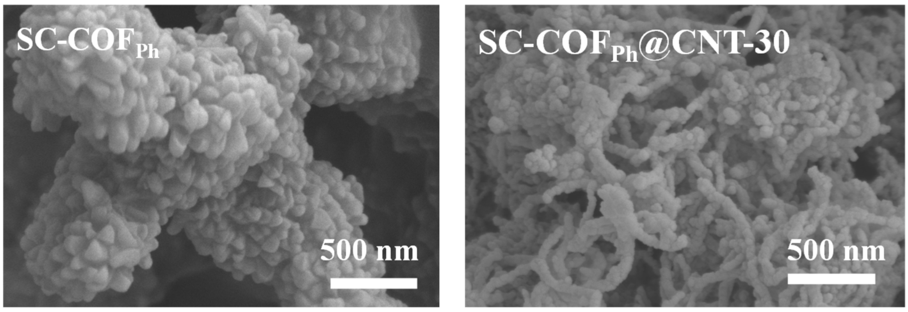

Supplementary Figure 55. SEM images of SC-COF$_{Ph}$ and SC-COF$_{Ph}$@CNT-30.

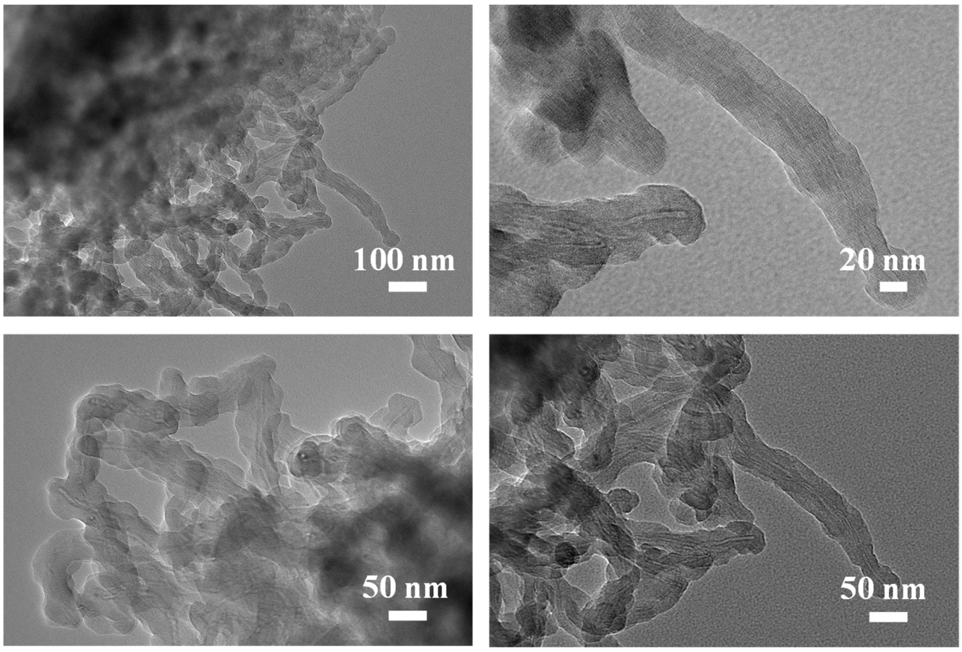

Supplementary Figure 56. TEM images of SC-COF$_{TSA}$@CNT-30.

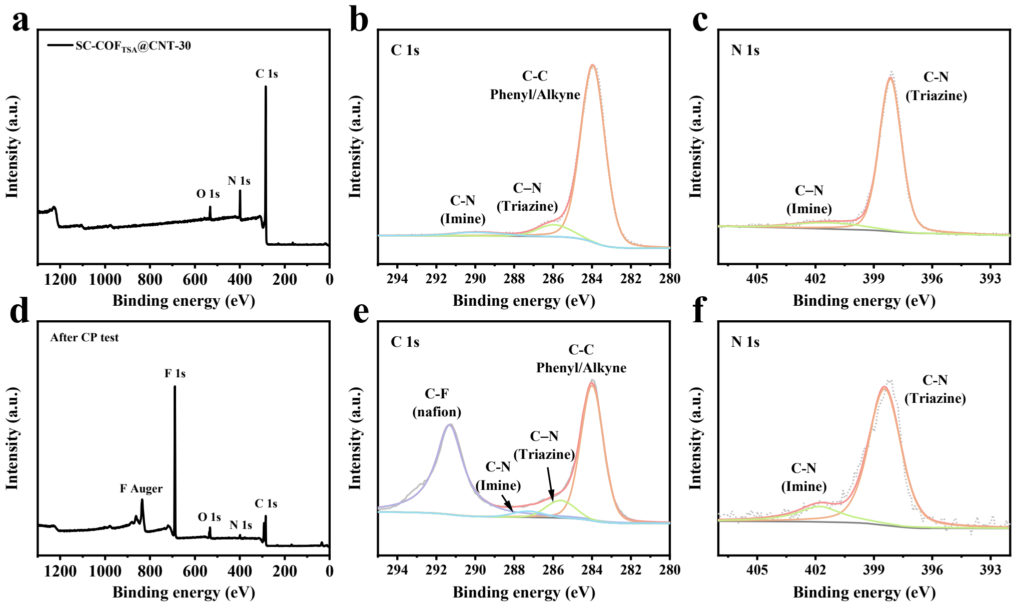

Supplementary Figure 57. XPS spectra of the original SC-COF$_{TSA}$@CNT-30 (a-c) and of the sample after chronopotentiometry (CP) test (d-f).

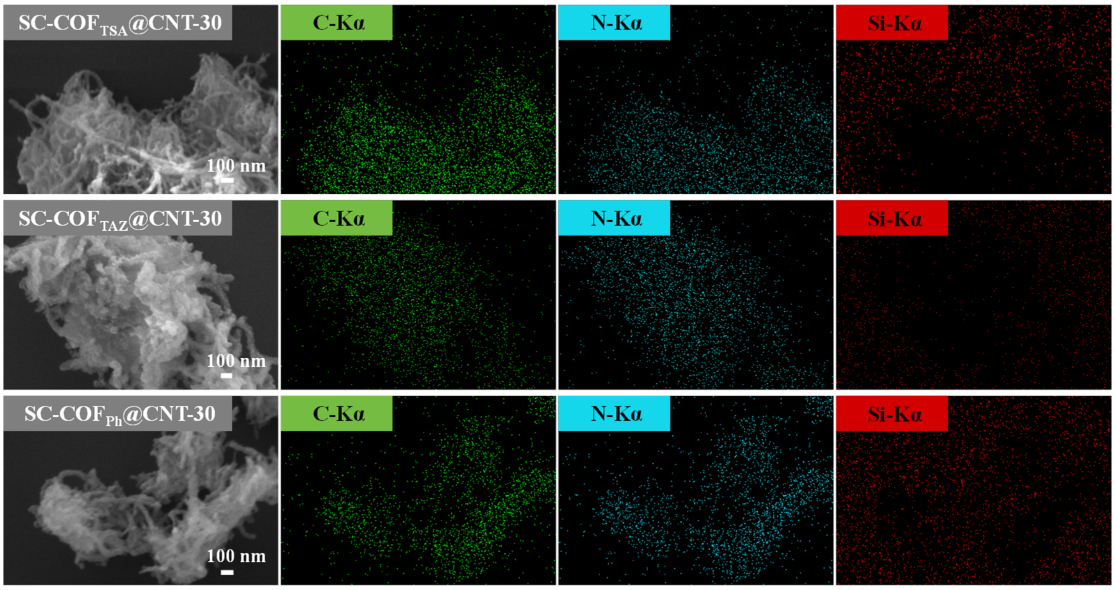

Supplementary Figure 58. EDS mapping images of SC-COF$_{TSA}$@CNT-30, SC-COF$_{TAZ}$@CNT-30 and SC-COF$_{Ph}$@CNT-30.

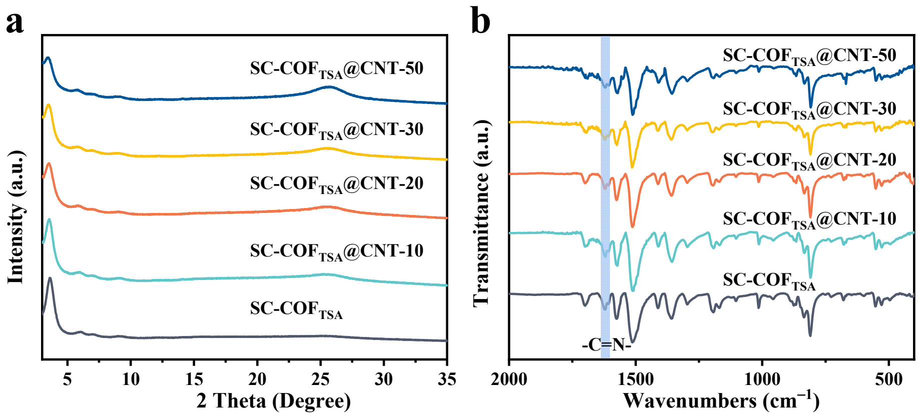

Supplementary Figure 59. XRD patterns and FT-IR spectra of SC-COF$_{TSA}$@CNT with different CNT loadings.

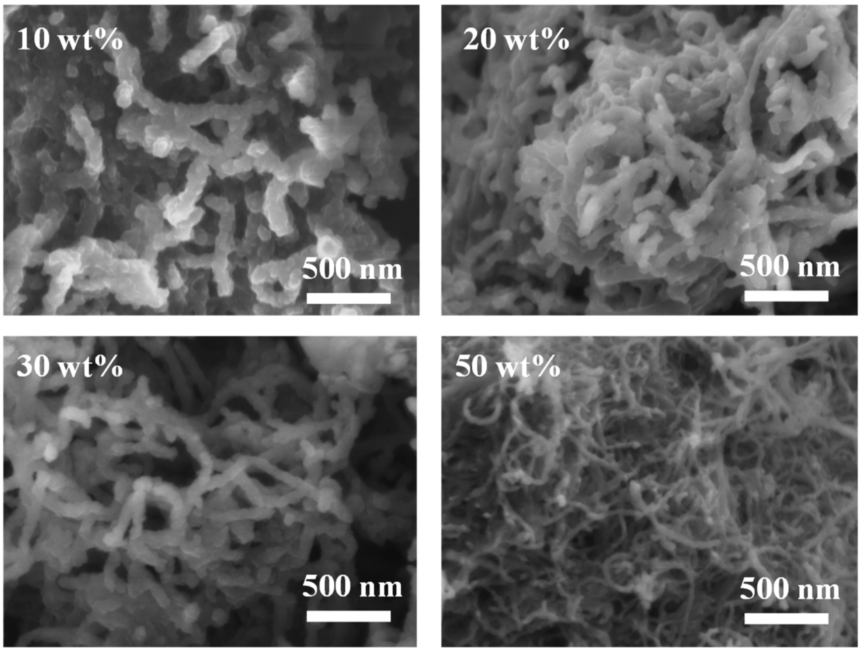

Supplementary Figure 60. SEM images of SC-COF$_{TSA}$@CNT with different CNT loadings.

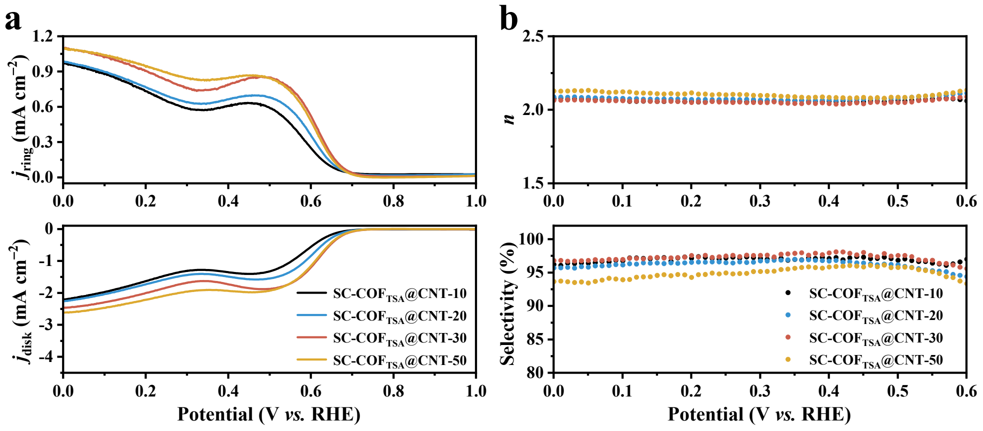

Supplementary Figure 61. Comparison of ORR performance of SC-COF$_{TSA}$@CNT with different CNT loadings.

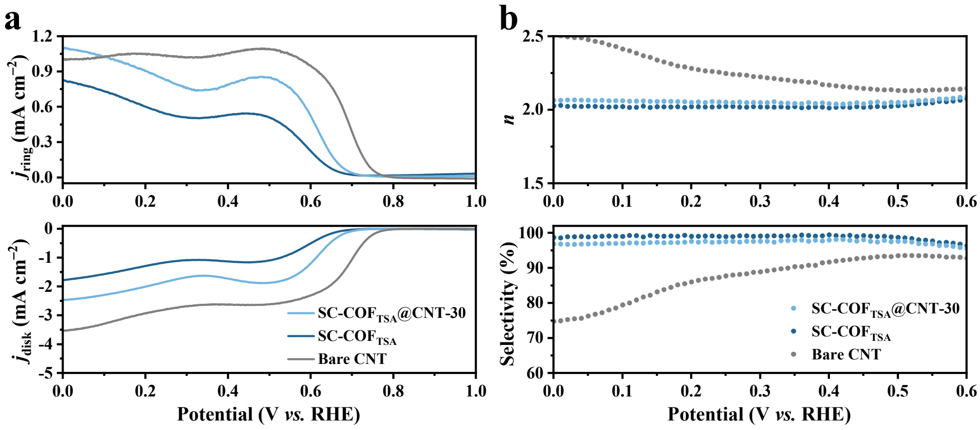

Supplementary Figure 62. Comparison of ORR performance of bare CNT, SC-COF$_{TSA}$ and SC-COF$_{TSA}$@CNT-30.

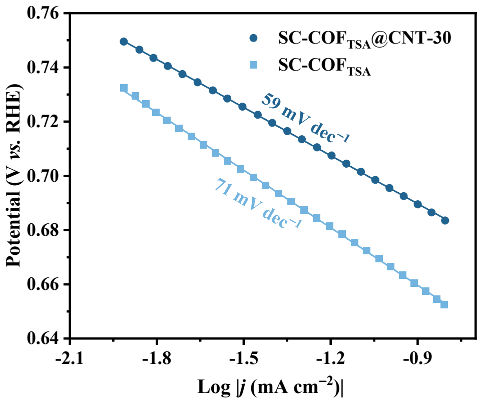

Supplementary Figure 63. Tafel slope of SC-COF$_{TSA}$ and SC-COF$_{TSA}$@CNT-30.

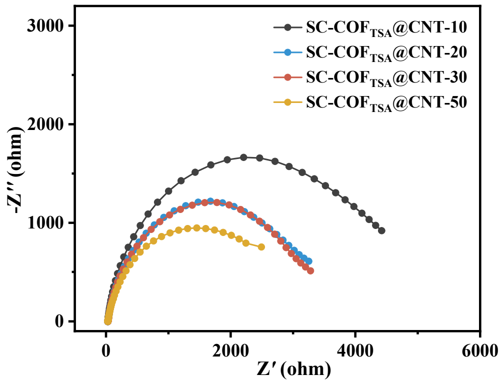

Supplementary Figure 64. EIS comparison of SC-COF$_{TSA}$@CNT with different CNT loadings.

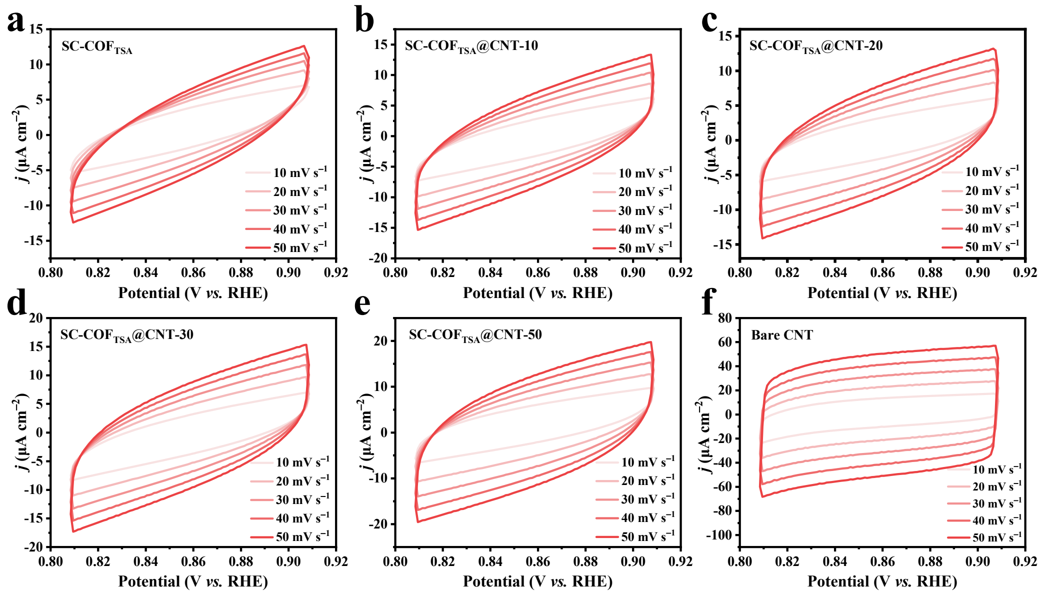

Supplementary Figure 65. CV curves of SC-COF$_{TSA}$@CNT with different CNT loadings.

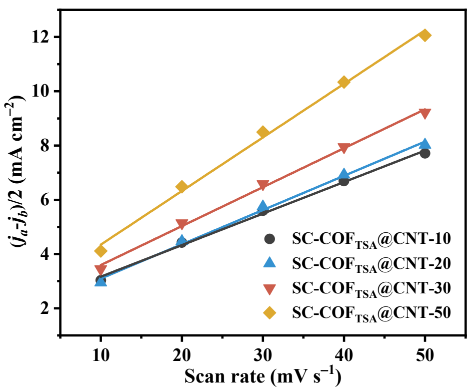

Sample	C_{dl} / μF cm^{-2}
SC-COF$_{TSA}$@CNT-10	28.8
SC-COF$_{TSA}$@CNT-20	31.2
SC-COF$_{TSA}$@CNT-30	35.5
SC-COF$_{TSA}$@CNT-50	48.9

Supplementary Figure 66. C_{dl} comparison of SC-COF$_{TSA}$@CNT with different CNT loadings.

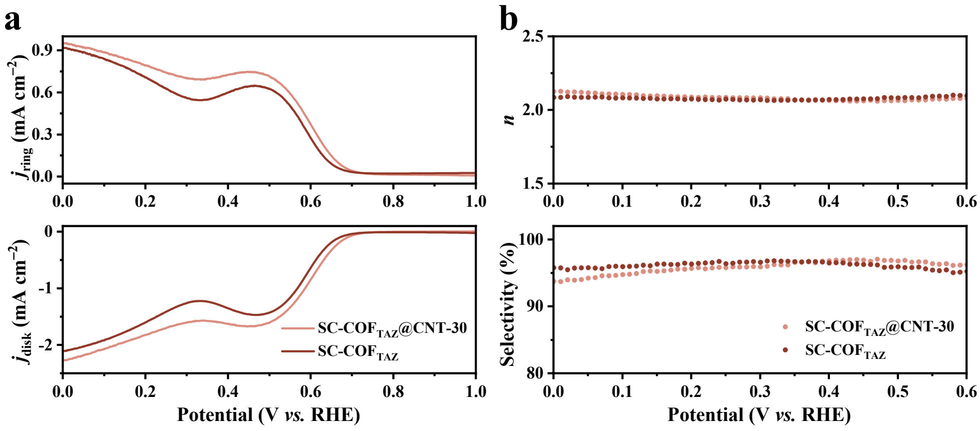

Supplementary Figure 67. The ORR performance comparison of SC-COF$_{TAZ}$ and SC-COF$_{TAZ}$@CNT-30.

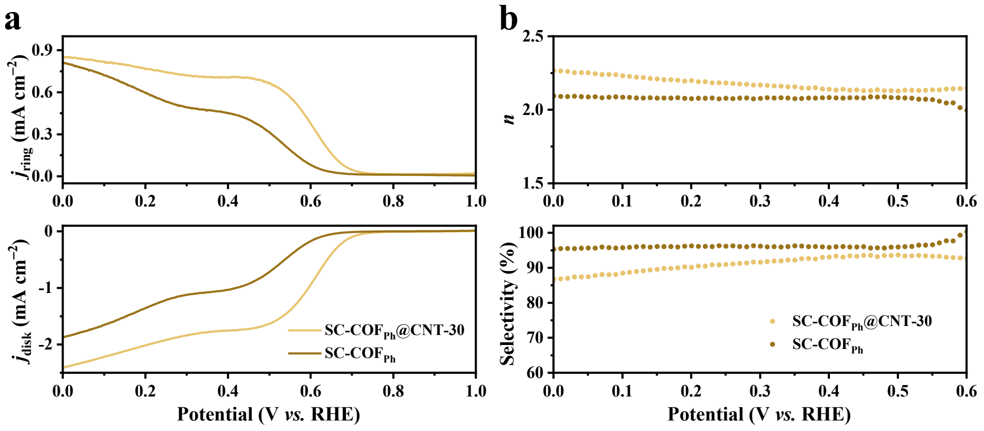

Supplementary Figure 68. The ORR performance comparison of SC-COF$_{Ph}$ and SC-COF$_{Ph}$@CNT-30.

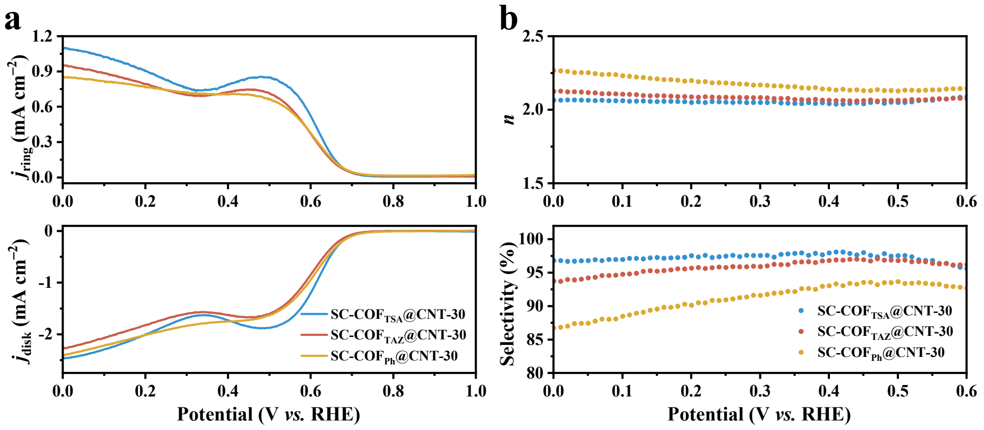

Supplementary Figure 69. The ORR performance comparison of SC-COF$_{TSA}$@CNT-30, SC-COF$_{TAZ}$@CNT-30 and SC-COF$_{Ph}$@CNT-30.

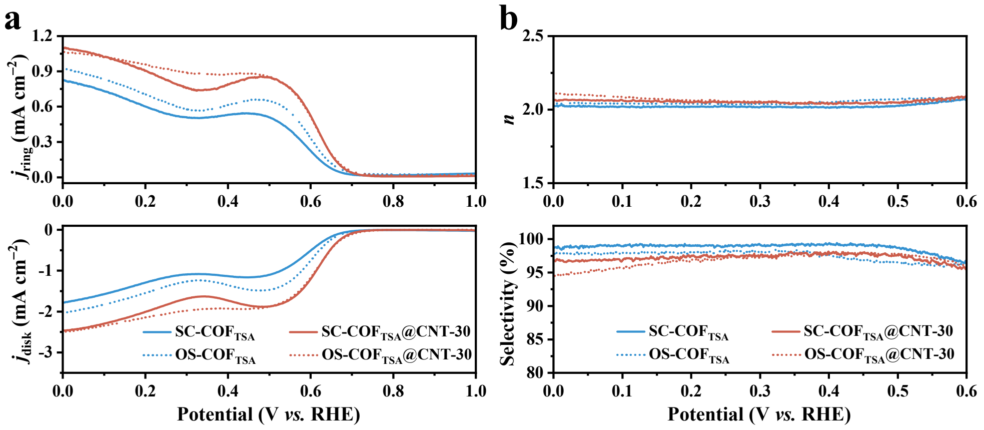

Supplementary Figure 70. ORR performance comparison of different synthesis methods.

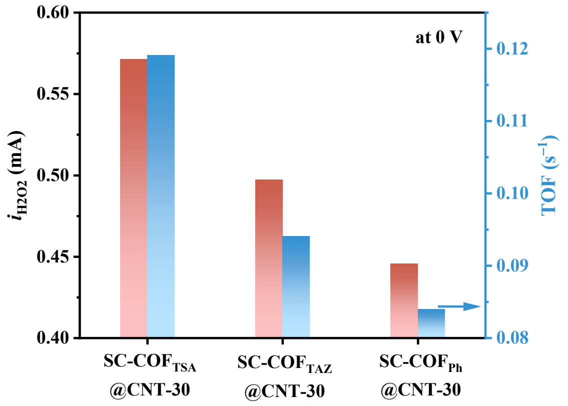

Supplementary Figure 71. The TOF of SC-COF$_{TSA}$@CNT-30, SC-COF$_{TAZ}$@CNT-30, and SC-COF$_{Ph}$@CNT-30.

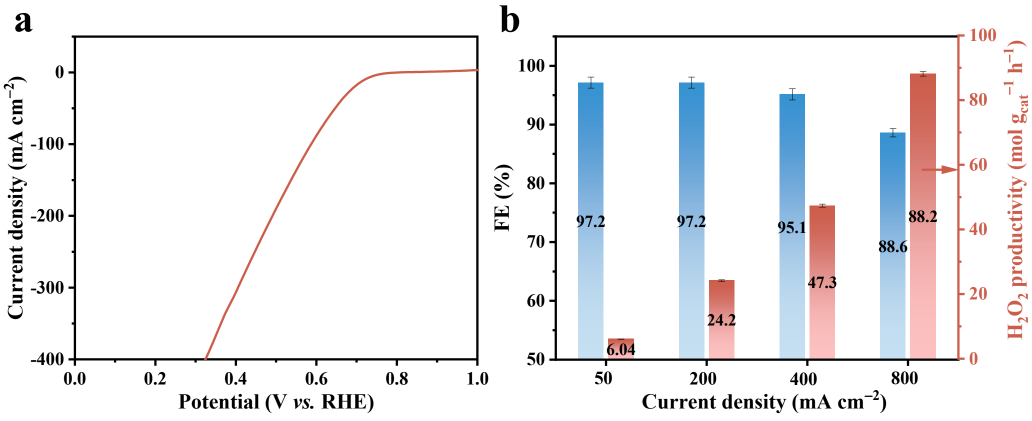

Supplementary Figure 72. LSV curve, FE, and H_2O_2 production rates for SC-COF$_{TAZ}$@CNT-30.

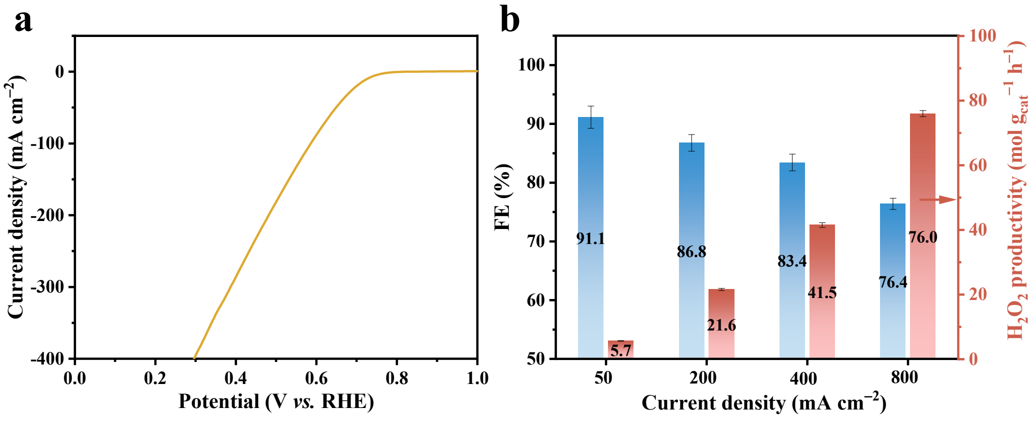

Supplementary Figure 73. LSV curve, FE, and H_2O_2 production rates for SC-COF$_{Ph}$@CNT-30.

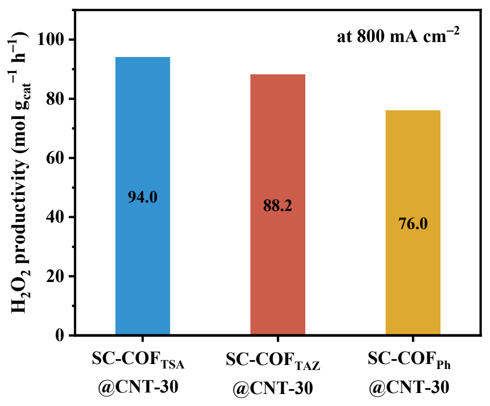

Supplementary Figure 74. H_2O_2 production rates for SC-COF$_{TSA}$@CNT-30, SC-COF$_{TAZ}$@CNT-30, and SC-COF$_{Ph}$@CNT-30 at 800 mA cm^{-2}.

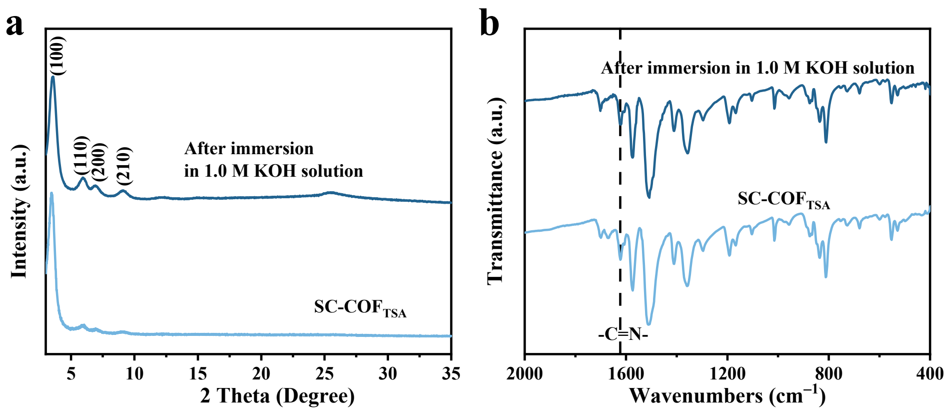

Supplementary Figure 75. XRD patterns and FT-IR spectra for SC-COF$_{TSA}$ before and after immersion in the 1.0 M KOH solution for 12 hours.

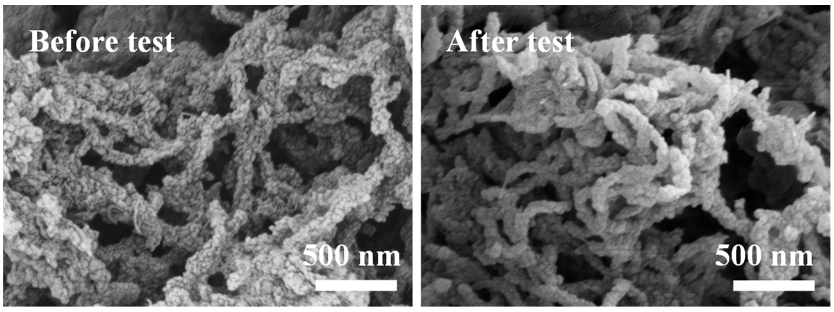

Supplementary Figure 76. SEM images of SC-COF$_{TSA}$@CNT-30 before and after electrochemical measurements.

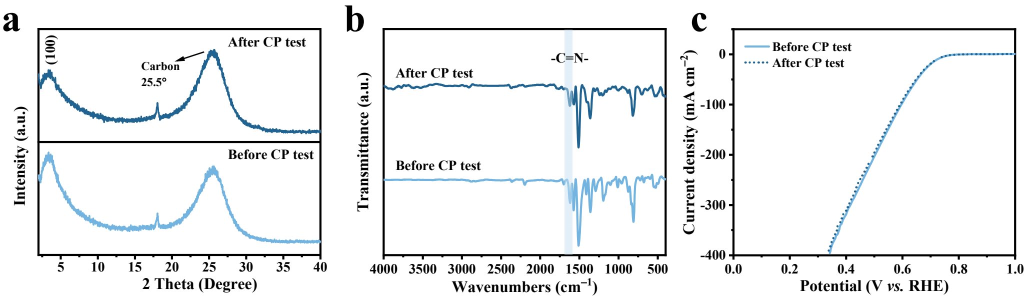

Supplementary Figure 77. XRD patterns, FT-IR spectra, and LSV curves of SC-COF$_{TSA}$@CNT-30 before and after electrochemical measurements.

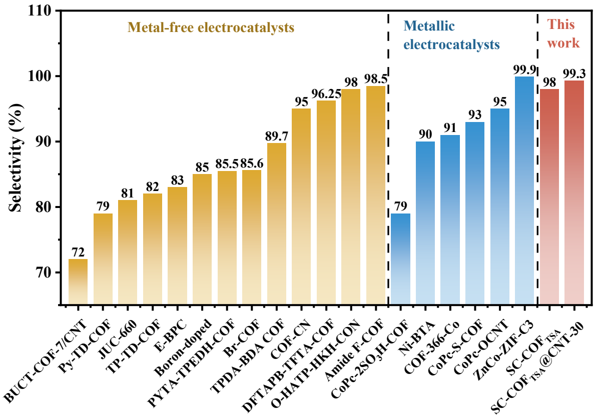

Supplementary Figure 78. Comparison of H_2O_2 selectivity with other reported literature under alkaline condition.

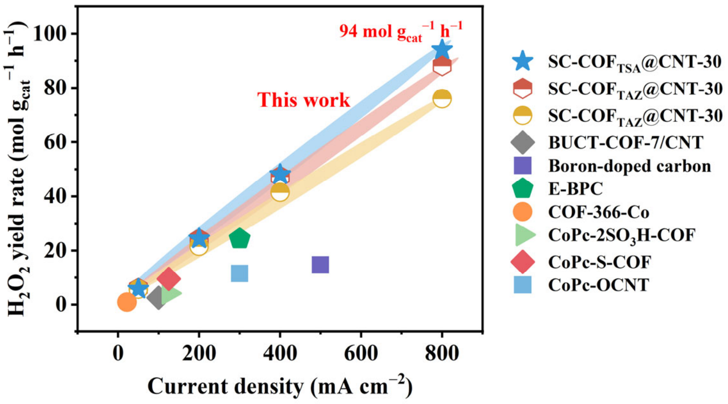

Supplementary Figure 79. Comparison of H_2O_2 yield rate with literature-reported values under alkaline condition.

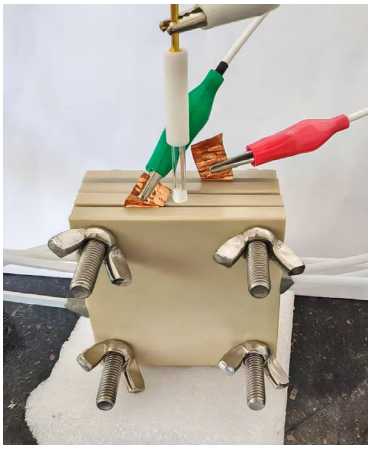

Supplementary Figure 80. The image of the Flow cell device.

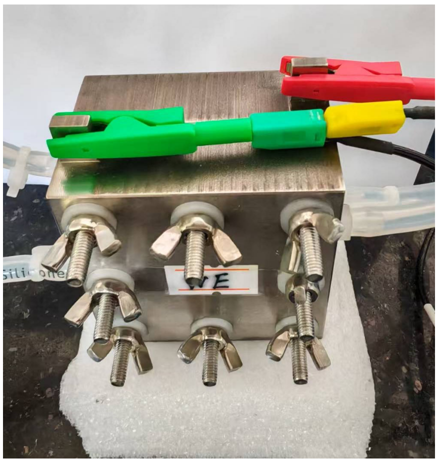

Supplementary Figure 81. The image of the membrane electrode assembly.

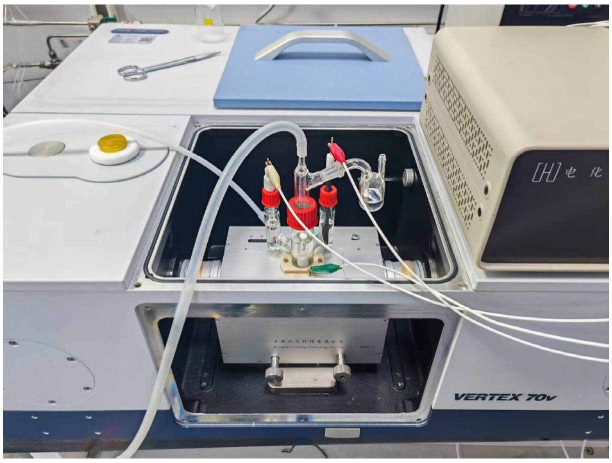

Supplementary Figure 82. The image of the *in-situ ATR-FTIR* characterization instrument。

Supplementary Table 1. Unit cell parameters for SC-COF_{TSA}, SC-COF_{TAZ}, and SC-COF_{Ph}.

	SC-COF_{TSA}	SC-COF_{TAZ}	SC-COF_{Ph}
Space group	P-6	P-6	P-6
Crystal system	Hexagonal	Hexagonal	Hexagonal
International tables number	174	174	174
a	30.5398 Å	26.0036 Å	26.2539 Å
b	30.5398 Å	26.0036 Å	26.2539 Å
c	3.4814 Å	3.4660 Å	3.4644 Å
α	90°	90°	90°
β	90°	90°	90°
γ	120°	120°	120°

Supplementary Table 2. Comparison of specific surface area of synthesized products at 5 min *vs.* 1 h synthesis.

	**5 min**	**1 h**
SC-COF_{TSA}	1249 $m^2 g^{-1}$	1178 $m^2 g^{-1}$
SC-COF_{TAZ}	1577 $m^2 g^{-1}$	2270 $m^2 g^{-1}$
SC-COF_{Ph}	1196 $m^2 g^{-1}$	959 $m^2 g^{-1}$
SC-COF_{TSA}@CNT-30	955 $m^2 g^{-1}$	1183 $m^2 g^{-1}$
SC-COF_{TAZ}@CNT-30	1166 $m^2 g^{-1}$	1674 $m^2 g^{-1}$
SC-COF_{Ph}@CNT-30	830 $m^2 g^{-1}$	833 $m^2 g^{-1}$

Supplementary Table 3. Comparison of yield of synthesized products at 5 min *vs.* 1 h synthesis.

	**5 min**	**1 h**
SC-COF_{TSA}	86.3%	88.7%
SC-COF_{TAZ}	79%	86.6%
SC-COF_{Ph}	70%	83.5%
SC-COF_{TSA}@CNT-30	92.9%	96.5%
SC-COF_{TAZ}@CNT-30	86.7%	89.7%
SC-COF_{Ph}@CNT-30	73.7%	87.5%

Supplementary Table 4. Comparison of specific surface areas of COFs synthesized by the supercritical CO_2 solvothermal method and the organic solvothermal method.

	**Supercritical CO_2 solvothermal**	**Organic Solvothermal**	**Reference**
**SC-COF_{TSA}**	1178 $m^2 g^{-1}$	410 $m^2 g^{-1}$	—
**SC-COF_{TAZ}**	2270 $m^2 g^{-1}$	TPT-COF[a] 1909.2 $m^2 g^{-1}$	*Angew. Chem. Int. Ed.* **2022**, *61*, e202208086
		COF-T3[b] 1596 $m^2 g^{-1}$	*J. Am. Chem. Soc.* **2024**, *146*, 30, 21025–21033
**SC-COF_{Ph}**	959 $m^2 g^{-1}$	TPT-TPB-COF[c] 836.9 $m^2 g^{-1}$	*Angew. Chem. Int. Ed.* **2022**, *61*, e202208086

The **abc** data are derived from the reference.

Supplementary Table 5. Band gap summarization of SC-COF based on different methods.

Band gap	SC-COF_{TSA}	SC-COF_{TAZ}	SC-COF_{Ph}
CV	2.82 eV	3.24 eV	3.16 eV

UV-Vis	2.57 eV	2.76 eV	2.67 eV
DFT	0.80 eV	1.09 eV	0.89 eV
E_{LUMO}-E_{HOMO}	3.11 eV	3.65 eV	3.46 eV

Supplementary Table 6. Electrochemical performance resistance measurement

Testing items	R (ohm)	Comp R (ohm)
COF_{TSA} (Fig. 3a)	28.0	23.8
COF_{TAZ} (Fig. 3a)	29.4	25.0
COF_{Ph} (Fig. 3a)	30.0	25.5
SC-COF_{TSA}@CNT-30 (Fig. 5d)	30.1	25.6
SC-COF_{TSA}@CNT-30 (Fig. 6b)	3.3	2.8
SC-COF_{TSA}@CNT-30 (Fig. 6e)	4.1	-
SC-COF_{TSA}@CNT-30 (Fig. 6g)	1.2	-

Supplementary Table 7. Summary and comparison of the H_2O_2 production performance of $2e^-$ ORR catalysts. (Operation conditions: alkaline solution)

**Categories of catalysts**	**Name of Catalysts**	**Synthesis Method**	**H_2O_2 Selectivity (%)**	**Faradaic Efficiency (%)**	**TOF (s^{-1})**	**H_2O_2 yield rate (mol g_{cat}^{-1} h^{-1})**	**Mass loading (mg cm^{-2})**	**Stability**	**Cell**	**Electrolyte (KOH)**	**Ref.**
**metal-free electro-catalysts**	SC-COF_{TSA}	Supercritical solvothermal, 80 ℃, 1 hour	99.3 (0.2 V_{RHE})	—	0.069 (0 V_{RHE})	—	0.15	/	Flow cell	1.0 M	This work
	SC-COF_{TSA}@CNT-30		98 (0.2 V_{RHE})	98.8 (400 mA cm^{-2})	0.119 (0 V_{RHE})	94 (800 mA cm^{-2})		Flow cell: 8 h (200 mA cm^{-2}) MEA: 50 h (400 mA cm^{-2})			
	PYTA-TPEDH-COF	Solvothermal, 120 ℃, 3 days	85.5 (0.2 V_{RHE})	80 (0.8 V_{RHE})	0.051 (/)	—	—	10 h (0.4 V_{RHE})	—	0.1 M	1

	BUCT-COF-7/CNT	One-pot synthesis, 130 ℃, 3 days	72 (0.4 V_{RHE})	70 (100 mA cm^{-2})	0.053 (0.7 V_{RHE})	2.5203 (100 mA cm^{-2})	0.5	24 h (0.3 V_{RHE})	H-type cell	0.1 M	2
	JUC-660	Solvothermal, 80 ℃, 7 days	81 (0.2 V_{RHE})	82.2 (0.1 V_{RHE})	0.069 (0.57 V_{RHE})	1.864 (0.1 V_{RHE})	0.5	85 h (40 mA cm^{-2})	Flow cell	0.1 M	3
	Py-TD-COF	Solvothermal, 120 ℃, 3 days	79 (0.2 V_{RHE})	—	—	0.218 (/)	/	16 h (0.55 V_{RHE})	H-type cell	0.1 M	4
	COF-CN	Solvothermal, 120 ℃, 3 days	95 (0.2 V_{RHE})	—	0.016 (/)	0.901 (0 V_{RHE})	0.2	28 h (/)	H-type cell	0.1 M	5
	TP-TD-COF	Solvothermal, 120 ℃, 3 days	82 (0.2 V_{RHE})	—	4 (/)	0.158 (0.55 V_{RHE})	—	20 h (0.55 V_{RHE})	H-type cell	0.1 M	6
	O-HATP-HKH-CON	Solvothermal	98 (0.2 V_{RHE})	93 (0.2 V_{RHE})	0.0134 (/)	0.139 (0.2 V_{RHE})	—	10 h (0.2 V_{RHE})	H-type cell	0.1 M	7

	Br-COF	Solvothermal, 120 ℃, 3 days	85.6 (0.2 V_{RHE})	—	0.2498 (0.5 V_{RHE})	—	—	10 h (0.3 V_{RHE})	—	0.1 M	8
	TPDA-BDA COF	Solvothermal, 120 ℃, 3 days	89.7 (0.2 V_{RHE})	86.7 (0.2 V_{RHE})	—	0.3765 (0.2 V_{RHE})	0.3	50 h (/)	—	0.1 M	9
	Amide F-COF	Solvothermal, two steps	98.5 (0 V_{RHE})	97.1 (200 mA cm^{-2})	0.155 (0.5 V_{RHE})	—	—	24 h (-0.4 V_{SCE})	—	0.1 M	10
	DFTAPB-TFTA-COF	RT, 5 days	96.25 (/)	71.8 (/)	0.0757 (0.5 V_{RHE})	0.253 (-0.4 V_{SCE})	—	3.47 h (-0.4 V_{SCE})	—	0.1 M NaOH	11
	Boron-doped carbon	Two-step synthesis (750 ℃)	85 (0.4 V_{RHE})	83.5 (0.4 V_{RHE})	45 (0.7 V_{RHE}) normalized by ECSA	14.72 (500 mA cm^{-2})	0.1	200 h (30 mA cm^{-2})	Flow cell	1 M	12
	E-BPC	Two-step synthesis (800 ℃)	83 (0.3 V_{RHE})	85 (100 mA cm^{-2})	—	24.3 (300 mA cm^{-2})	0.25-0.5	100 h (100 mA cm^{-2})	Flow cell	1 M	13

	COF-366-Co	Solvothermal, 120 ℃, 3 days	91 (0.3 V_{RHE})	79 (22 mA cm^{-2})	1.79 (0.4 V_{RHE})	0.909 (22 mA cm^{-2})	0.35	3 h (22 mA cm^{-2})	H-type cell	0.1 M	14
	CoPc-2SO_3H-COF	Solvothermal, 120 ℃, 3 days	79 (0.2 V_{RHE})	90 (125 mA cm^{-2})	—	4.2 (125 mA cm^{-2})	0.16	20 h (125 mA cm^{-2})	Flow cell	0.1 M	15
**metallic electro-catalysts**	CoPc-S-COF	Solvothermal, 100 ℃, 7 days	93 (0.2 V_{RHE})	75 (0.15 V_{RHE}), 91 (0.45 V_{RHE})	—	9.5 (125 mA cm^{-2})	0.48	20 h (125 mA cm^{-2})	Flow cell	1 M	16
	Ni-SAC	Three-step synthesis	—	89 (0.5 V_{RHE})	0.78 (0.6 V_{RHE})	7.30 (-1.7 V_{RHE})	0.4	35 h (-1.7 V_{RHE})	Flow cell	0.1 M	17
	ZnCo-ZIF-C3	CTAB-assisted solvothermal method,	~100 (0.2 V_{RHE})	95 (20 mA cm^{-2})	—	4.35 (60 mA cm^{-2})	0.61	100 h (0 V_{RHE})	—	0.1 M	18

		100 ℃, 6 hours									
	Ni-BTA	Ammonia-assisted coordination polymerization	90 (0.3 V_{RHE})	90 (0.2 V_{RHE})	2.27 (-0.42 V_{RHE})	34 (-0.4 V_{RHE})	0.1	40 h (0.2 V_{RHE})	Flow cell	1 M	19
	CoPc-OCNT	Template pyrolysis method	95 (0 V_{RHE})	98 (100 mA cm^{-2})	32 (300 mA cm^{-2})	11.527 (300 mA cm^{-2})	0.5	30 h (200 mA cm^{-2})	Flow cell	1 M	20

3. Supplementary References

1. An, S. et al. One-dimensional covalent organic frameworks for the 2e⁻ oxygen reduction reaction. *Angew. Chem. Int. Ed.* **62**, e202218742 (2023).
2. Zhang, Y. et al. Multicomponent synthesis of imidazole-linked fully conjugated 3D covalent organic framework for efficient electrochemical hydrogen peroxide production. *Angew. Chem. Int. Ed.* **62**, e202314539 (2023).
3. Wang, R. et al. Structural modulation of covalent organic frameworks for efficient hydrogen peroxide electrocatalysis. *Angew. Chem. Int. Ed.* **63**, e202410417 (2024).
4. Huang, S. et al. Linkage engineering in covalent organic frameworks as metal-free oxygen reduction electrocatalysts for hydrogen peroxide production. *Appl. Catal. B* **340**,123216 (2024).
5. Xu, X. et al. Regulating the activity of intrinsic sites in covalent organic frameworks by introducing electro-withdrawing groups towards highly selective H_2O_2 electrosynthesis. *Nano Today* **49**, 101792 (2023).
6. Huang, S. et al. Covalent organic frameworks with molecular electronic modulation as metal free electrocatalysts for efficient hydrogen peroxide production. *Small Struct.* **4**, 2200387 (2023).
7. Yang, S. et al. Rational design of edges of covalent organic networks for catalyzing hydrogen peroxide production. *Appl. Catal. B. Environ.* **298**, 120605 (2021).
8. Li, X. et al. Micro-modulation of linkers of covalent organic frameworks as catalysts for 2e⁻ oxygen reduction reaction. *Appl. Catal. B* **344**, 123611 (2024).
9. Liu, J. et al. Rational design two- or four-electron reaction pathway covalent organic frameworks for efficient and selective electrocatalytic hydrogen peroxide production. *Angew. Chem. Int. Ed.* **64**, e202424720 (2025).
10. Jimenez-Duro, M. et al. Robust amide-linked fluorinated covalent organic framework for long-term oxygen reduction reaction electrocatalysis. *Small* **20**, 2402082 (2024).
11. Martinez-Fernandez, M. et al. Scalable synthesis and electrocatalytic performance of highly fluorinated covalent organic frameworks for oxygen reduction. *Angew. Chem. Int. Ed.* **62**, e202313940 (2023).
12. Xia, Y. et al. Highly active and selective oxygen reduction to H_2O_2 on boron-doped carbon for high production rates. *Nat. Commun.* **12**, 4225 (2021).

13. Byeon, A. et al. CO_2-derived edge-boron-doped hierarchical porous carbon catalysts for highly effective electrochemical H_2O_2 production. *Appl. Catal. B. Environ.* **329**, 122557 (2023).
14. Liu, C. et al. Intrinsic activity of metal centers in metal-nitrogen-carbon single-atom catalysts for hydrogen peroxide synthesis. *J. Am. Chem. Soc.* **142**, 21861–21871 (2020).
15. Jiang, R. et al. Hydrophilic phthalocyanine covalent organic frameworks for enhanced electrocatalytic H_2O_2 production. *Chem. Eng. J.* **489**, 151232 (2024).
16. Zhi, Q. et al. Dithiine-linked metalphthalocyanine framework with undulated layers for highly efficient and stable H_2O_2 electroproduction. *Nat. Commun.* **15**, 678 (2024).
17. Sun, Y. et al. Boosting electrochemical oxygen reduction to hydrogen peroxide coupled with organic oxidation. *Nat. Commun.* **15**, 6098 (2024).
18. Zhang, C. et al. Crystal engineering enables cobalt-based metal-organic frameworks as high-performance electrocatalysts for H_2O_2 production. *J. Am. Chem. Soc.* **145**, 7791–7799 (2023).
19. Sang, Z. et al. Internal hydrogen-bond enhanced two-electron oxygen reduction reaction for *π-d* conjugated metal-organic framework to H_2O_2 synthesis. *Nat. Commun.* **16**, 4050 (2025).
20. Cao, P. et al. Metal single-site catalyst design for electrocatalytic production of hydrogen peroxide at industrial-relevant currents. *Nat. Commun.* **14**, 172 (2023).
