# Supplementary material for: Supercritical CO2-assisted rapid synthesis of covalent organic framework-based electrocatalyst for efficient two-electron oxygen reduction reaction
Source: Nat Commun. 2025 Oct 8;16:8963. doi: 10.1038/s41467-025-64901-1 (PMC12508161; doi:10.1038/s41467-025-64901-1)
Supplement: Supplementary file 2 — Description of Additional Supplementary Files [file 41467_2025_64901_MOESM2_ESM.pdf]

Description of Additional Supplementary Files

Supplementary Data 1. Atomic coordinates after the model optimization of SC-COF_{TSA}.

Supplementary Data 2. Atomic coordinates after the model optimization of SC-COF_{TAZ}.

Supplementary Data 3. Atomic coordinates after the model optimization of SC-COF_{Ph}.
